# Understanding the Ultrafast Crystallization Kinetics of Donor–Acceptor Semiconducting Polymers

**DOI:** 10.1021/jacs.6c07715

**Published:** 2026-08-11

**Authors:** Josep Tent-Pérez, Francisco Blanco-Vázquez, Jorge L. Olmedo-Martínez, Alberto Peinador Veiga, Matteo Sanviti, Marián Prada-Cortés, Antoine Curé, Eugenio Solla, Bernat Mundet, Cyril Aumaître, Xabier Rodriguez-Martinez, Renaud Demadrille, Jaime Martin

**Affiliations:** † 73077 Universidade da Coruña, Campus Industrial de Ferrol, CITENI, Campus de Esteiro, 15403 Ferrol, Spain; ‡ IRIG-SyMMES, Université Grenoble Alpes/CEA/CNRS/Grenoble INP, 38000 Grenoble, France; § Universidade da Coruña, Servicios de apoyo a la investigación (SAI), Campus de Elviña, s/n, 15071 A Coruña, Spain; ∥ Catalan Institute of Nanoscience and Nanotechnology (ICN2), Campus UAB, Bellaterra, 08193 Barcelona, Catalonia, Spain; ⊥ Oportunius Programme, Axencia Galega de Innovación, Xunta de Galicia, C/Rúa Airas Nunes s/n, 15702 Santiago de Compostela, Spain

## Abstract

The crystallization of donor–acceptor semiconducting polymers occurs on subsecond time scales at elevated temperatures (>300 °C), preventing a rigorous kinetic description using conventional experimental approaches. Here, we use a single-step fast scanning calorimetry (FSC) methodology that enables investigating the fast isothermal crystallization kinetics of advanced donor–acceptor semiconducting polymers such as PTQ10 and D18. This approach overcomes the intrinsic limitations of the indirect “by-step” method and provides reliable kinetic data within tenths of a second. Furthermore, we demonstrate that a modified version of the Malkin model, which we also introduce here, successfully captures the entire kinetic evolution and provides important information about how crystallization develops in these polymers. For example, we observe two well-differentiated crystallization regimes as a function of the crystallization temperature. A newly introduced geometrical parameter of the kinetic model reveals that the distinct crystallization kinetics result in different crystalline morphologies, which were further confirmed by X-ray diffraction, melting analysis, and electron microscopy. Our analysis suggests that crystallization proceeds via a nearly instantaneous formation of a dense population of nanoscopic crystallites and is effectively governed by a single dominant kinetic step that must therefore be strongly linked to nucleation. However, self-nucleation experiments reveal that this process does not conform to the classical nucleation behavior typically observed in semicrystalline polymers. These findings allow establishing a new framework for the understanding and control of the solid-state microstructure of semicrystalline polymers.

## Graphical abstract

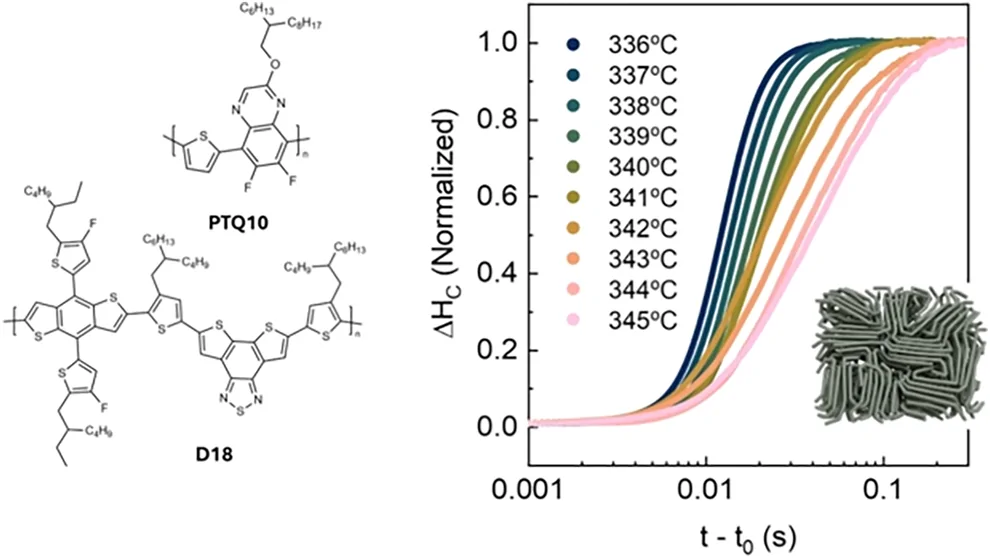

## 1 Introduction

Organic semiconductors, particularly conjugated polymers, have attracted significant attention for applications in organic electronics due to lightweight, conformable (flexible), and large-area device fabrication.^1,2^ It is now well-recognized that the performance of these devices is critically linked to the solid-state microstructure of the constituent polymers, as the optoelectronic properties of these materials are highly sensitive to molecular packing. For example, charge transport benefits from an increased degree of π–π orbital overlapping and reduced energetic disorder, which are typically achieved in regions with compact, periodic molecular packing, e.g., crystals.^3,4^ Because crystals are generated during the solidification of the material via the crystallization process, it is of paramount importance to understand how crystallization occurs in this special class of polymers.

It is well-known that donor–acceptor semiconducting polymers do not feature lamellar crystals like typical semicrystalline polymers, but they arrange into a type of low-order structure commonly known as solid mesophase.^5^ Hence, under typical device operating temperatures, these polymers generally display a biphasic solid-state microstructure combining mesophase and more disordered (amorphous-like) regions. The mesophase has been proposed to be a layered structure, where solid-like layers formed by π-stacked conjugated backbone segments alternate with liquid-like, flexible layers composed of aliphatic side chains. Within π-stacked layers, the molecular packing tends to contain high degrees of disorder.^6–8^ Despite the substantial progress in characterizing the structural order in these polymers, the mechanisms governing the mesophase formation remain poorly understood (We would like to note that in order to use the traditional nomenclature of the field, here, we will refer to “crystallization kinetics” and “crystals” or “crystallites” to refer to the kinetics of formation of the mesophase and the mesophase domains, respectively).

Crystallization kinetic studies under isothermal conditions have been the fundamental approach for elucidating how crystallinity develops in polymer materials. Monitoring the time-dependent evolution of the degree of crystallinity from the melt provides insights into the pathways/mechanisms of the crystallization process. The interpretation of the crystallization kinetics has traditionally been framed in terms of crystal nucleation and crystal growth steps. Therefore, classical models/theories such as the classical nucleation theory and the Avrami framework have provided the basis for describing crystallization mechanisms, and their polymer-specific extensions, including the Hoffman–Lauritzen theory, have captured other aspects of chain-folded lamellar growth.^9–11^ We would like to note that the importance of melt crystallization analysis to understand the crystallization process in semiconducting polymers is discussed in the Supporting Information (Figure S1).

Isothermal crystallization kinetics can be followed, e.g., by differential scanning calorimetry (DSC) via the analysis of the heat exchanged during isothermal crystallization. This method is typically known as the continuous or direct or one-step method. Hence, DSC stands out as the standard method for determining the crystallization kinetics of semicrystalline polymers, and studies on, e.g., poly­(3-(2́-ethyl)­hexylthiophene) (P3EHT) have demonstrated that these classical methodologies can also be applied to simple semiconducting polymers.^12^ However, modern, more advanced semiconducting polymers exhibit high melting temperatures (often >300 °C)^13^ at which thermal degradation of the material occurs, preventing the standardized DSC analysis. Partially solving this issue, the use of fast scanning calorimetry (FSC) has emerged as a powerful tool to study the crystallization kinetics of these types of materials.^14,15^ Thanks to the fast heating/cooling rates that can be applied in FSC, i.e., >10^3^ °C/s, degradation can be minimized. However, the application of the one-step protocol in the FSC equipment is strongly limited by the reduced sample size and the resulting small calorimetric signal during the crystallization step. Therefore, to the best of our knowledge, this methodology has been so far applied on very few occasions and solely to simple semicrystalline polymers, including polyolefins, polyamides, and the like.^16–19^ Conversely, the few studies reporting the crystallization kinetics of advanced donor–acceptor conjugated polymers have used the so-called “by-step” approach, where the polymer is subjected to steps of variable length at a certain isothermal crystallization temperature. Then, the evolution of the melting enthalpy is monitored as a function of the step length. This method was used by Luo et al. to investigate the crystallization kinetics of diketopyrrolopyrrole (DPP)-based donor–acceptor polymers and poly­(3-alkyltiophenes) (P3AT) polymers.^20,21^ However, the “by-step” methodology can be tedious and challenging to apply to materials whose melting temperatures overlap with their degradation temperatures (as it is frequently the case for semiconducting polymers) because multiple repeated melting/crystallization cycles are required.

Here, we show how the continuous, direct method can be applied in FSC to investigate the ultrafast isothermal crystallization kinetics of high-performance donor–acceptor polymers such as PTQ10 and D18 in a single step (chemical structures are shown in Figure 1a). This approach overcomes the limitations of the “by-step” approach mentioned above and enables the acquisition of reliable kinetic data within fractions of a second. Moreover, we show that a generalized version of the Malkin model accurately describes the full crystallization kinetics and provides insights into the crystallization process. The analysis reveals two distinct crystallization regimes depending on the crystallization temperature. Interestingly, a newly introduced geometrical parameter of the Malkin model reveals that these regimes lead to different crystalline morphologies, consistent with results from wide-angle X-ray diffraction (WAXS), melting analysis, and high-resolution transmission electron microscopy (HRTEM). Overall, our results indicate that crystallization proceeds through a nearly instantaneous formation of a dense population of nanoscopic crystallites governed by a single dominant kinetic step.

**Figure 1:**
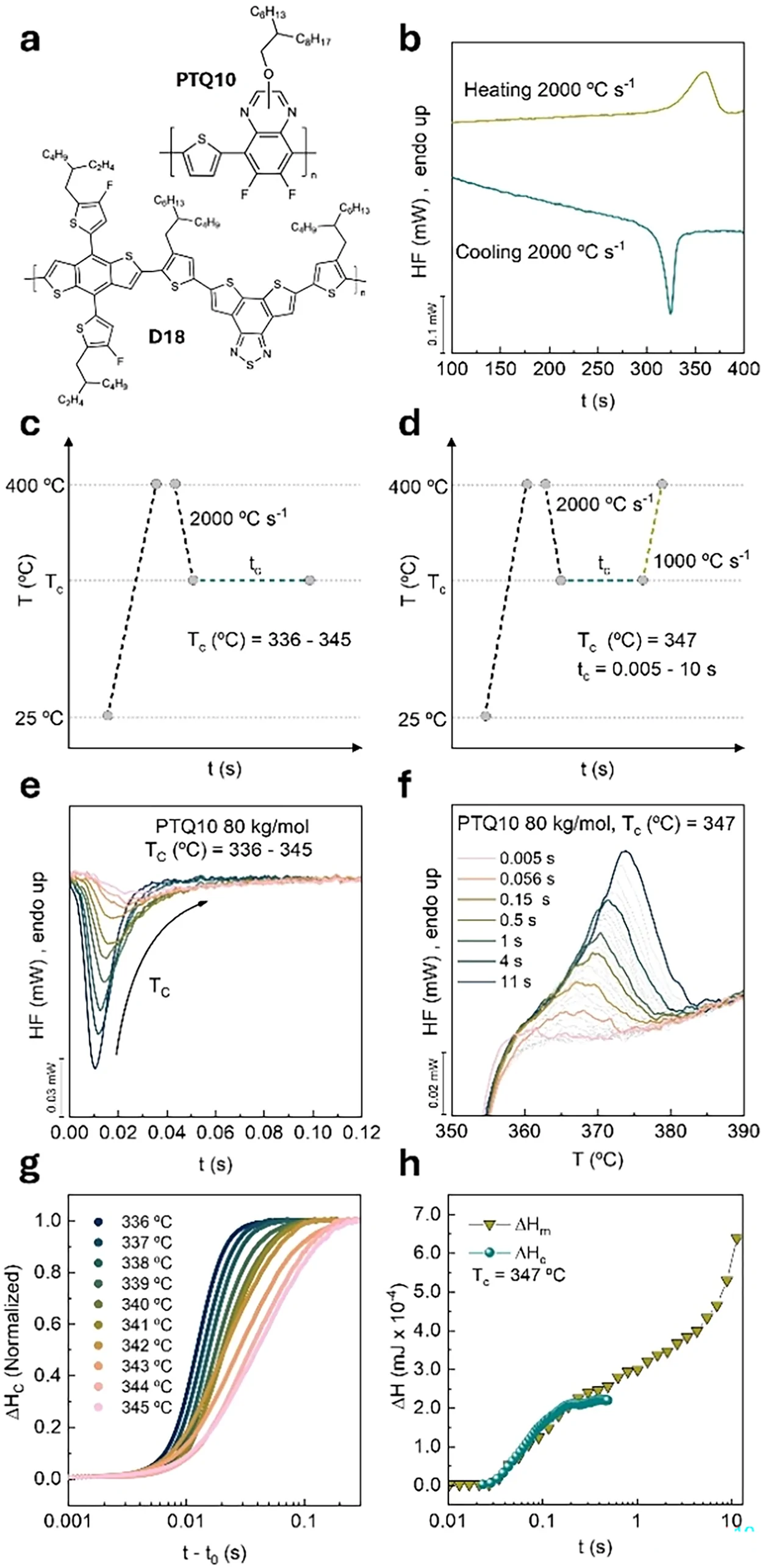
(a) Chemical structures of PTQ10 and D18. (b) Representative FSC heating and cooling trances (at 2000 °C) for PTQ10. (c) Thermal protocol applied for the investigation of isothermal crystallization kinetics in a single step (i.e., the direct method). (d) Thermal protocol applied for the investigation of isothermal crystallization kinetics “by steps”. (e) Crystallization exotherms for PTQ10 crystallized at different crystallization temperatures, *T*_*c*_ *s* (from 336 to 345 °C). (f) Evolution of the melting peaks of crystals formed at *T_c_* = 347 °C during isothermal steps of the length indicated. (g) Evolution of the enthalpy change, Δ*H_c_*, thereby the crystalline conversion, α, during the isothermal crystallization of PTQ10 at different *T_c_s.* (h) Comparison between melting enthalpy, Δ*H_m_*, and crystallization enthalpy, Δ*H_c_*, for PTQ10 isothermally crystallized at *T_c_* = 347 °C.

## 2 Results

The crystallization of donor–acceptor semiconducting polymers is an extremely fast process. The crystallization of most of the polymers,^22–24^ and many inorganic and hybrid materials,^25,26^ is suppressed when they are cooled from the melt to a temperature below their glass transition temperature, *T_g_*, at cooling rates of the order of 10^4^ °C/s. However, this is not the case for donor–acceptor semiconducting polymers. Shown in Figure 1b are the heat flow rate (HF) vs temperature, *T*, fast scanning calorimetry, FSC, traces obtained during heating and cooling PTQ10 (with a weight-average molar mass (*M*_w_) of 80 kg mol^–1^ and a dispersity, *Đ*, of 2.5) at −2000 °C/s. The intense and well-defined exothermic peak that can be observed in the cooling trace (blue line) is due to an extensive crystallization of the material within the 0.025 s period in which the temperature of the material decreases from 350 to 300 °C. The melting of these crystals gives rise to an intense endothermic signal in the subsequent heating scan (green curve). Such a fast crystallization rate together with the fact that crystallization in these polymers occurs frequently above 300 °C (i.e., at temperatures at which the material’s thermal degradation is also fast) prevents the analysis of the isothermal crystallization kinetics by standard methods, e.g., by regular differential scanning calorimetry, DSC. However, the extraordinary fast sampling rate (10 kHz) and the extreme heating/cooling rates that can be applied in FSC (up to 10^5^ °C/s), which, on the one hand, allow reaching the isothermal crystallization temperature before the crystallization has started and on the other hand minimize the time spent at high temperatures (where the degradation rate is fast), allow probing the isothermal crystallization of donor–acceptor semiconducting polymers in a single isothermal step. We note that, so far, isothermal crystallization of semiconducting polymers has been investigated “by steps”,^15,20,27^ i.e., measuring the enthalpy of melting, Δ*H_m_*, of crystals formed during isothermal steps of different lengths (see the thermal protocol of the “by-step” method in Figure 1d).

In order to investigate the crystallization kinetics of PTQ10 in a single step, the thermal protocol depicted in Figure 1c was applied. Briefly, samples were first heated well above their melting temperatures to erase any previous thermal history. Then, they were rapidly cooled, i.e., at −2000 °C/s, to the target isothermal crystallization temperature, *T_c_*, at which samples were kept for 0.1–0.3 s, while the heat exchanged with the environment (i.e., the *HF* signal) was recorded. Suitable *T_c_* values were selected from a cooling scan conducted at the same cooling rate (see Figures 1b and S2a of the Supporting Information). This thermal treatment is detailed in the Experimental Section contained in the Supporting Information. We must note that all investigated samples are bulk, droplet-like pieces. Exemplary sample masses were, e.g., 109 ng and 157 ng for PTQ10 and D18, respectively. These mass values are calculated from sample volumes (exemplary diameter and height values were 100 and 22 μm, respectively, for the PTQ10 sample, and were 95 and 33 μm, respectively, for D18) and considering a density of 1.1 g/mL.

Figure S2b of the Supporting Information shows exemplary raw *HF vs t* curves obtained during the isothermal step at different *T_c_* s. These data suggest that the exothermic signal due to the crystallization of PTQ10 is in the low-*t* region superimposed to a second process, likely associated with the thermal stabilization of the system (i.e., the sample plus the calorimeter). To withdraw this contribution, we analyzed the *HF* at an intentionally high *T_c_*, in this case at 360 °C (Figure S2 of the Supporting Information), at which crystallization cannot occur, and thereby, the *HF* signal recorded solely reflects the thermal stabilization. When this contribution is subtracted from the raw *HF* signals recorded in the relevant *T_c_* range, the exothermic *HF* curves shown in Figure 1e are obtained (see an example of the baseline subtraction in Figure S2c of the Supporting Information). The cumulative integration of these exotherms as a function of time, *t*, provides the evolution of the enthalpy change during crystallization, Δ*H_c_*, and thereby the advance of the crystalline conversion, α (Figure 1g). Note that isothermal crystallization occurs less than 0.1 s for all *T_c_* analyzed, highlighting once again the fast liquid–solid transition of these polymers.

To validate our methodology, the kinetics of PTQ10 were compared with those obtained by the “by-step methodology” (see the thermal protocol in Figure 1d). Figure 1f features the evolution of the melting peaks of crystals formed at *T_c_* = 347 °C within a series of isothermal steps of variable length, *t*_c_. The integral value of the melting peaks at each *t*_c_ provides the Δ*H_m_* (for each *t*_c_), which also accounts for the fraction of the phase-transformed material and thus can be compared with the Δ*H_c_* from the “direct experiments”. As can be observed in Figures 1h and S3 of the Supporting Information, both experimental protocols exhibit the same trend and enthalpy values, at least during the initial 0.5 s, in which the crystallization is already saturated (according to the “direct experiment”). This demonstrates the usefulness of the direct kinetic analysis by FSC. We would like to note that the rationale for the continuous increase of the Δ*H_m_* beyond 0.5 s in the “by-step” method is unknown and needs to be investigated in the future. However, from the thermogravimetric and Fourier transform infrared spectroscopy (FTIR) data included in Figure S4 of the Supporting Information, we hypothesize that it could be due to a further molecular packing of polymers as a consequence of side chain cleavage due to thermal degradation during long isothermal steps.

In order to further test the validity of the methodology, the thermal protocol was applied to a well-studied, reference polymer, such as poly­(ethylene oxide) (PEO). As shown in Figure S5 and Table S1 of the Supporting Information, the crystallization kinetics of PEO can be adequately investigated with the direct method by FSC and the obtained kinetic parameters, e.g., the Avrami indices (*n*), agree well with those previously reported in the literature.^28,29^ Additionally, to ensure that no thermal degradation occurs during the single-step isothermal experiments, melting curves before and after the isotherms were compared for both PTQ10 and D18 polymers. As shown in Figure S6, both curves perfectly overlap, indicating the absence of thermal degradation.

Motivated by the validation of our experimental approach, we extended our investigation to another high-performance semiconducting polymer for organic photovoltaics applications, the so-called D18 polymer (with a *M*_w_ of 63 kg mol^–1^and *Đ* = 1.9), which compared to PTQ10 has a significantly bulkier repeating unit (see the chemical structure of D18 in Figure 1a). Isothermal crystallization kinetic data for D18 are shown in Figure S7 of the Supporting Information. We would like to note that, as shown in Figure S8 of the Supporting Information, the crystallization kinetics of these polymers is not affected by the size/thickness of samples, which rules out effects from inefficient thermal transport across the samples during the cooling step prior to isothermal crystallizations.

Having obtained reliable crystallization kinetic data for PTQ10 and D18, we tried to gain an understanding of how the crystallization of donor–acceptor semiconducting polymers occurs. For that, we analyzed our results in the context of existing crystallization models, starting from the Avrami model, which is commonly used to describe the crystallization kinetics of semicrystalline polymers. The model yields the following expression

1 $$\alpha \left(t\right)=1-\exp\left({-k\left(t-t_{0}\right)}^{n}\right)$$

where *k* is the overall crystallization rate constant, *t*_0_ is the induction time or the time for primary nucleation (before growth has started), and *n* is the Avrami index, which accounts for how the nucleation and crystal growth process depend on time, as it is further detailed in the Supporting Information.

It must be pointed out that the Avrami model usually describes well the initial part of the crystallization of semicrystalline polymers, which includes the primary nucleation and the free growth of crystalline features (e.g., spherulites).^30,31^ Likewise, fittings of isothermal crystallization curves of PTQ10 and D18 to the Avrami equation fail at α > 30% (see Figure 2a,2b). Hence, in order to ensure a reliable evaluation of the kinetic parameters, the Avrami equation was fitted in the α-range between 3 and 20%.

**Figure 2:**
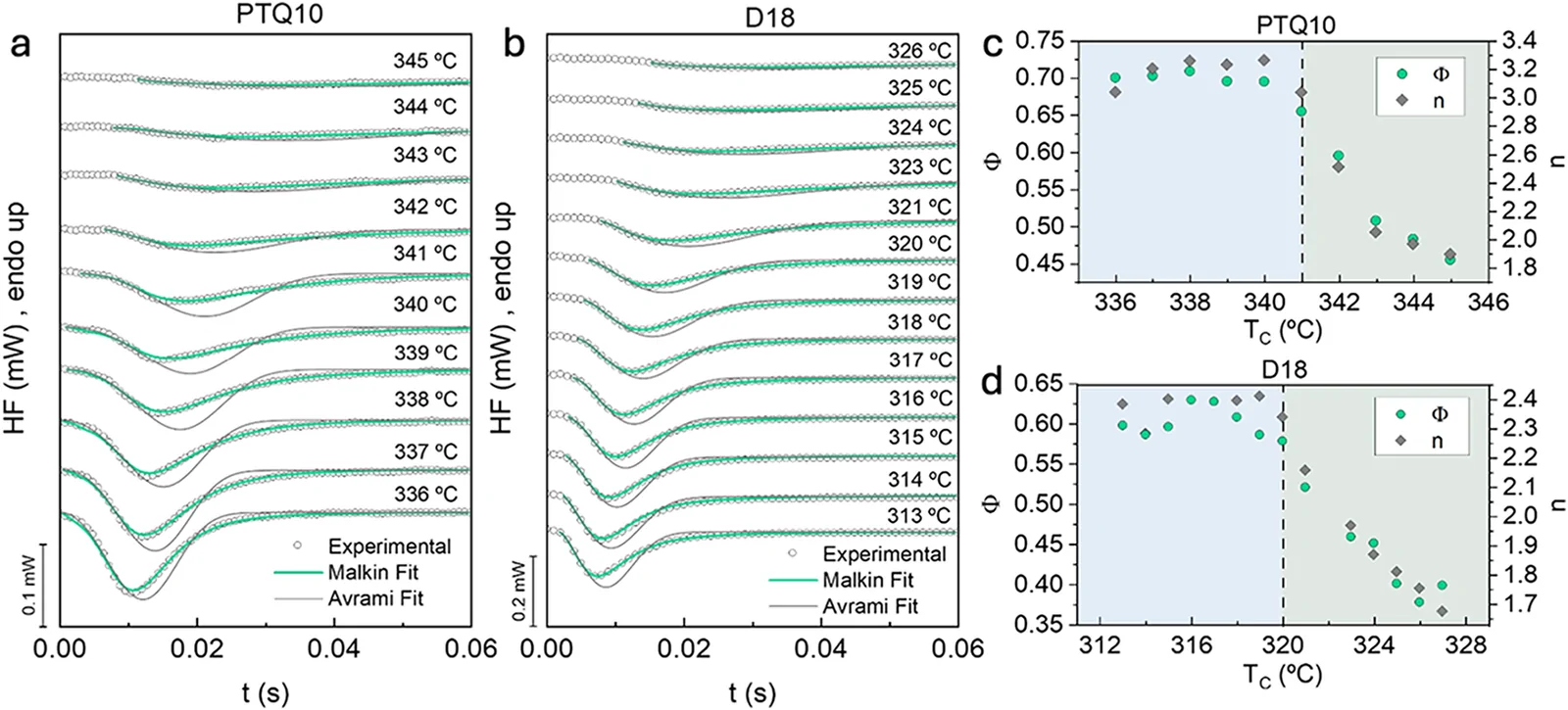
Isothermal crystallization exotherms at various *T_c_* s for PTQ10 (a) and D18 (b). Hollow circles are experimental data; gray lines are fits to the Avrami model and green lines are fits to the Malkin model. (c, d) Evolution of the Avrami exponent (*n*) and Malkin parameter (Φ) expressed as a function of *T_c_* s for PTQ10 and D18, respectively.

The calculated *n*-values for PTQ10 and D18 ranged from ∼2 to ∼3 and from ∼1 to ∼2, respectively (see Table 1). Interestingly, the analysis reveals a common *n vs T_c_* trend in both materials, with a marked decrease of *n* at a critical *T_c_* of 341 °C for PTQ10 and 320 °C for D18 (see Figure 2c,2d). Above these critical *T_c_* s, *n* parameters in both materials remain relatively constant, whereas they decrease sharply at higher *T_c_* s. However, because *n*-values gather contributions from nucleation and growth, they are difficult to interpret.

**Table 1:** Fitting Parameters of the Avrami and Malkin Models for Isothermally Crystallized PTQ10 and D18

	PTQ10							D18					
	Avrami		Malkin					Avrami		Malkin			
*T* (°C)	*k* (s^–*n*^)	*n*	*k*_1_(s^–1^)	*k*_2_^′^ (^−1^)	Φ	*n*_M_	*T* (°C)	*k* (s^–*n*^)	*n*	*k*_1_(s^–1^)	*k*_2_^′^ (s^–1^)	Φ	*n*_M_
**336**	4.4 × 10^05^	3.04	1.0 × 10^–04^	261.5	0.70	1.15	**313**	8.6 × 10^04^	2.38	1.1 × 10^–06^	316.0	0.60	1.48
**337**	6.9 × 10^05^	3.21	3.0 × 10^–06^	237.5	0.70	1.24	**314**	4.8 × 10^04^	2.28	1.2 × 10^–05^	297.4	0.59	1.45
**338**	5.9 × 10^05^	3.26	8.9 × 10^–07^	218.4	0.71	1.31	**315**	7.7 × 10^04^	2.40	4.7 × 10^–07^	286.1	0.60	1.33
**339**	4.0 × 10^05^	3.23	5.4 × 10^–06^	188.3	0.70	1.51	**316**	1.3 × 10^05^	2.56	4.6 × 10^–05^	288.5	0.63	1.47
**340**	3.3 × 10^05^	3.26	9.3 × 10^–05^	169.4	0.70	1.62	**317**	8.9 × 10^04^	2.52	3.3 × 10^–07^	268.4	0.63	1.48
**341**	1.3 × 10^05^	3.04	1.3 × 10^–05^	141.8	0.66	1.58	**318**	4.2 × 10^04^	2.40	2.7 × 10^–07^	235.5	0.61	1.42
**342**	1.4 × 10^04^	2.51	1.4 × 10^–06^	113.7	0.60	1.64	**319**	2.6 × 10^04^	2.41	1.2 × 10^–06^	203.4	0.59	1.39
**343**	1.4 × 10^03^	2.05	2.6 × 10^–08^	73.7	0.51	1.67	**320**	1.7 × 10^04^	2.34	2.0 × 10^–07^	178.5	0.58	1.40
**344**	7.7 × 10^02^	1.96	4.7 × 10^–05^	58.5	0.48	1.67	**321**	5.7 × 10^03^	2.16	2.8 × 10^–08^	124.3	0.52	1.50
**345**	4.7 × 10^02^	1.89	4.0 × 10^–07^	47.4	0.45	1.62	**323**	1.6 × 10^03^	1.97	2.7 × 10^–09^	81.0	0.46	1.57
**-**	**-**	**-**	**-**	**-**	**-**	**-**	**324**	8.8 × 10^02^	1.87	5.5 × 10^–06^	68.9	0.45	1.55
**-**	**-**	**-**	**-**	**-**	**-**	**-**	**325**	6.3 × 10^02^	1.81	7.0 × 10^–09^	58.4	0.40	1.51
**-**	**-**	**-**	**-**	**-**	**-**	**-**	**326**	4.8 × 10^02^	1.75	2.8 × 10^–10^	51.1	0.38	1.38
**-**	**-**	**-**	**-**	**-**	**-**	**-**	**327**	3.1 × 10^02^	1.67	7.2 × 10^–08^	50.1	0.40	1.48

Therefore, in order to gain further insights into the crystallization behavior of these materials, we decided to investigate an alternative kinetic model: the Malkin model. Inspired by chemical reaction kinetics, Malkin et al. developed a kinetic framework that describes the crystallization process through rate equations.^32^ Here, crystal nucleation and growth contributions to the crystallization rate are considered separately, in such a way that the fraction of crystallized material, α, is given by

2 $$\frac{d\alpha }{dt}=\left(k_{1}+k_{2}S\left(\alpha \right)\right){\left(1-\alpha \right)}^{n_{M}}$$

where *k*_1_ and *k*_2_ are independent constants related to the nucleation and autocatalytic crystal growth rate, respectively, *n*_*M*_ is a constant analogous to a chemical reaction order, and *S*(α) is a function that accounts for the surface area over which the crystal formation occurs. Therefore, *S*(α) is related to the geometry of the growing crystals. To find an analytical solution for eq ([Disp-formula eq2]), Malkin et al. considered that *n*_*M*_ equals to 1 and that *S*(α) takes the linear form *S*(α) = *D*α. This approximation turned out to be rather valid for flexible polymers that crystallize with spherulitic growth.^33,34^ Although the spherulitic surface-to-volume-ratio would escalate with a factor of 2/3 (if a pure geometrical ratio is considered), the phenomenological description of the active crystallization interface pushes the scale factor to 1, likely due to interlamellar amorphous regions and lamellar branching. However, it is well-known that donor–acceptor semiconducting polymers do not tend to exhibit spherulites.^5^ Thus, for our analysis, we established that the surface area over which crystal formation occurs has the general form *S*(α) = *D*α^Φ^, where Φ is a constant that captures how the surface area involved in crystal formation scales with the fraction of material that has already transformed. For example, when Φ = 0, the surface area remains constant during crystallization; if 0 < Φ < 1, the surface area grows slower than transformation, e.g., in compact or layered growth; if Φ = 1, the surface area grows linearly with a transformed fraction; when Φ > 1, the surface area grows faster than the volume of the transformed phase, e.g., in dendritic or fractal-like growth. Thus, the modified Malkin equation takes thus the following form

3 $$\frac{d\alpha }{dt}=\left(k_{1}+k_{2}^{\prime }\alpha^{\Phi }\right){\left(1-\alpha \right)}^{n_{M}}$$

where *k*_2_^′^ = *k*_2_ *D*. The full development of the modified Malkin model is described in the Supporting Information. Numerical solution of eq 3 with the Runge–Kutta optimization algorithm “RK45” from the SciPy Python package^35,36^ allows us to fit the entire crystallization curves of PTQ10 and D18 for all *T_c_* s analyzed, as can be seen in Figure 2a,2b. Fitting parameters used are summarized in Table 1. All standard errors and R^2^ coefficients are reported in Table S2 of the Supporting Information.

With a new set of parameters available for analysis, the new Malkin model allows a more detailed study of the crystallization behavior in these materials. Thus, we analyzed the obtained Malkin parameters one by one, starting with the geometrical parameter Φ. Tellingly, the dependence of Φ with *T_c_* for both materials exhibits a nearly identical trend as the Avrami exponent, *n*, with critical *T_c_* s of 341 °C for PTQ10 and 320 °C for D18, suggesting two crystallization regions with different kinetics as a function of *T_c_* (see Figure 2c,2d).

Figure 2c,2d and Table 1 show that Φ acquired values of ∼0.7 in the low-*T_c_* region and of ∼0.45 in the high-*T_c_* region for PTQ10, whereas the Φ-parameter for D18 crystallization was ∼0.6 in the low-*T_c_* region and ∼0.4 in the high-*T_c_* region. To further interpret the values obtained from the Φ parameter, we analyzed other semicrystalline polymers with a known morphology, e.g., poly­(ethylene oxide) (PEO), polycaprolactone (PCL), and poly­(1,6-hexanediol) (P6) and the semiconducting polymer poly­(3-hexylthiophene) (P3HT). As shown in Figures S9–11 of the Supporting Information, PEO and PCL, which typically exhibit spherulitic (i.e., 3D) morphology,^37,38^ featured Φ values ranging from 0.8 to 1. On the other hand, P6 and P3HT, which are known to develop axialites,^39^ exhibit Φ-values between 0.6 and 0.8, as shown in Tables S3–S5 of the Supporting Information. Based on these observations, a Φ between 0.8 and 1 can be associated with three-dimensional (3D) growth; 0.6 < Φ < 0.8 refers to a 2D axialitic growth; a Φ between 0.4 and 0.6 is connected with the growth of two-dimensional (2D) planar structures; and last, a Φ < 0.4 associates with one-dimensional (1D) structures. Hence, in the low-*T_c_* region, PTQ10 falls in the Φ-range that is associated with the growth of 3D features, whereas at high *T_c_s*, it falls in the Φ range associated with 2D structures. For the case of D18, low *T_c_s* result in typical Φ values of 2D structures and then fall into the Φ range associated with 1D features at high *T_c_s*. Interestingly, calculated Avrami indices, *n*, would agree with the interpretation above if an instantaneous nucleation is assumed, as *n* values of ∼3 and ∼2 are calculated for PTQ10 in the low- and high*-T_c_* regimes, respectively, and those measured for D18 are ∼2 and ∼1, respectively (see Table 1).

Motivated by this finding, we investigated by wide-angle X-ray diffraction (WAXS), FSC, and atomic force microscopy (AFM) whether the different crystallization kinetics found above and below the critical *T_c_* s result in different crystalline features. We must highlight that this structural analysis needed to be performed on polymers supported onto Flash DSC chip sensors, as isothermal crystallizations can only be conducted, in a controlled manner, using Flash DSC equipment. While AFM does not show significant morphological differences between samples crystallized in the low- and high*-T_c_* regions (see Figure S12 of the Supporting Information), WAXS and melting analysis suggest the development of crystals with different characteristics. Shown in Figure S13 of the Supporting Information are the transmission WAXS results (area detector images in the reciprocal space are shown). Figure 3a–3d shows the detailed views of the corresponding integrated line profiles (i.e., intensity, *I*, vs scattering vector, *q*) regions where the main diffraction peaks, i.e., the (100) and (010) peaks, show up. The ratio of the integrated intensities of the (100) and the (010) peaks, *A*_(100)_ and *A*_(010)_, respectively, provides information about the tendency of the material to pack along the [100] and [010] directions. Therefore, *A*_(100)_/*A*_(010)_ is related to the morphology of the crystalline lattice. Tellingly, the dependence of *A*_(100)_/*A*_(010)_ with *T_c_* exhibits, once again, the same critical *T_c_* values as *n* and Φ parameters, as can be observed in Figure 3g,3i. This suggests that the different crystallization kinetics found in the *T_c_* regions below and above the critical *T_c_s* relate to the formation of crystals with different morphologies.

**Figure 3:**
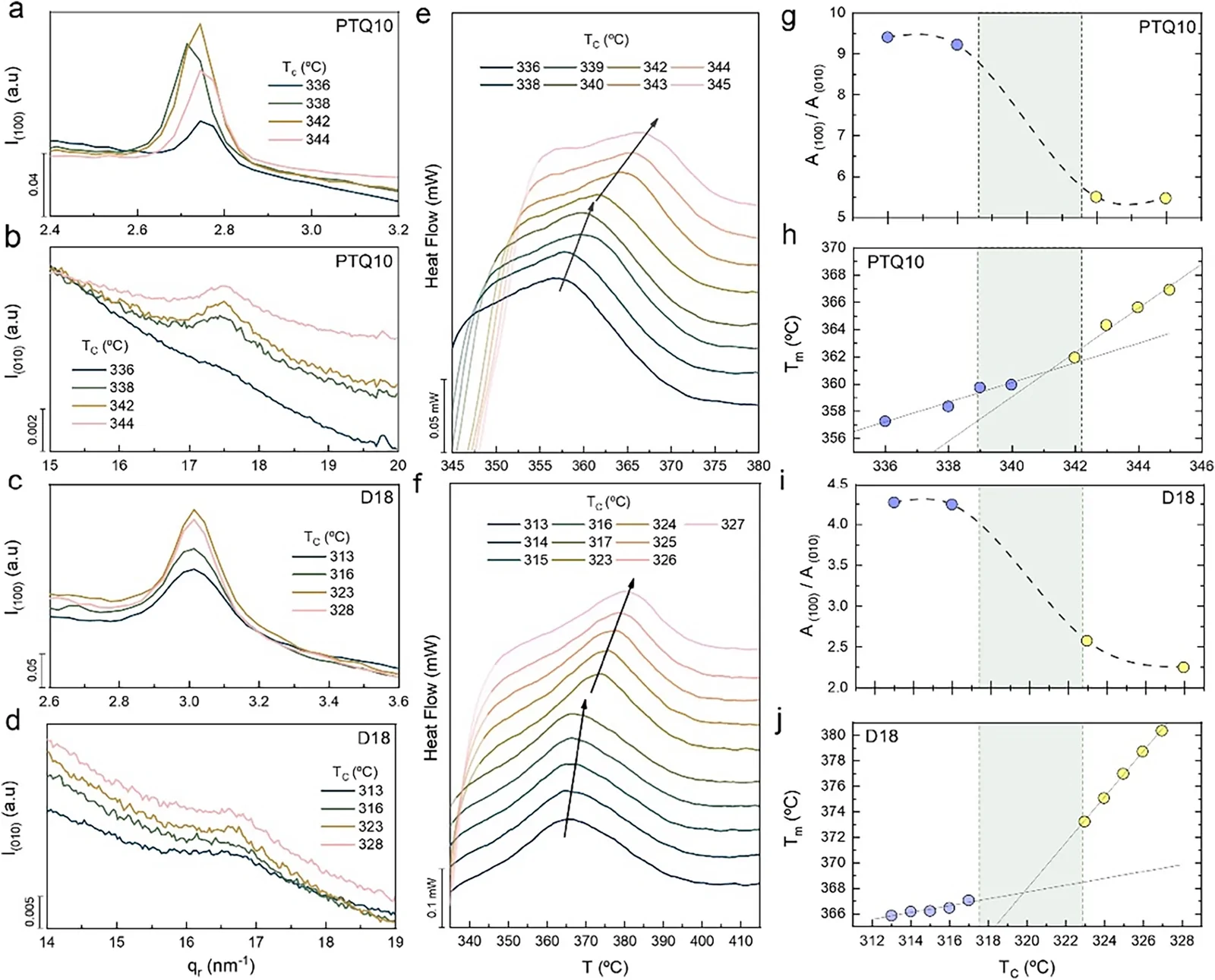
Linecuts with diffraction intensities of (100) lamellar and (010) π–π stacking peaks for PTQ10 (a, b) and D18 (c, d). Flash DSC heating traces of PTQ10 (e) and D18 (f) isothermally crystallized at different temperatures, 336 to 345 °C and 313 to 326 °C, respectively. The ratio of the integrated intensities of the (100) and (010) peaks, *A*_(100)_ and *A*_(010)_, for PTQ10 (g) and D18_(i)_, crystallized at different temperatures, 336, 338, 342, and 344 °C and 313, 316, 323, and 328 °C, respectively. Melting temperature evolution of PTQ10 (h) and D18 (j), at different crystallization temperatures. All thermograms were represented with the endothermic events upside.

This conclusion is further supported by the analysis of the melting temperatures of isothermally crystallized materials, *T_m_* s. It is well-known that the *T*_*m*_ of polymer crystals directly correlates with the free energy of the crystal, and thereby with the size of the crystallite.^40^ Indeed, in regular semicrystalline polymers, *T*_*m*_ is close linked to the lamellar thickness, i.e., the crystallite size along the chain direction within the lamellae. We argue that for donor–acceptor semiconducting polymers, the situation might be slightly different, as they do not form in lamellar crystals and exhibit nanoscale size along crystallographic directions other than the chain direction. Therefore, the crystallite size along directions other than the extended chain direction can also impact *T*_*m*_. In any case, *T*_*m*_ must be equally—or even more strongly—associated with crystallite size in semiconducting polymers. Figure 3e,f shows the FSC melting peaks for PTQ10 and D18 materials isothermally crystallized at the indicated *T_c_s*. Clearly, the shift of the melting peak as a function of *T_c_* exhibits two well-differentiated trends. This can be more clearly seen in Figure 3h,3j, in which the *T*_*m*_s (peak temperatures) are plotted against *T_c_.* Here again, we observe two markedly different behaviors below and above a *T_c_* of 341 °C for PTQ10 and a *T_c_* 320 °C for D18, thereby further suggesting that crystals with different morphological characteristics, in this case free energy/size, are developed in those *T_c_* regions.

Further information about the crystallization mechanisms/kinetics of these semiconducting polymers can be extracted from the independent rate constants of the nucleation and the growth steps, *k*_*1*_ and *k*_*2*_ *´*, respectively. Strikingly, we found that the *k*_*2*_ *´* values were 6 to 9 orders of magnitude larger than those of *k*_1_ at all temperatures and for both polymers. This large difference of rate constants seems to indicate that the overall crystallization rate, and thereby the crystallization kinetics, is governed by a single crystallization step, unlike the other polymers studied here (i.e., PEO, P6, P3HT, and PCL), which exhibit smaller differences between *k*_1_ and *k*_2_´ (see Tables S3–S5 of the Supporting Information).

According to the classical interpretation of Malkin *k*_*1*_ and *k*_*2*_´ rate constants, our analysis suggests that the nucleation rate in these semiconducting polymers is much lower than the growth rate. It must be noted that such kinds of kinetic scenarios typically result in the formation of few and large crystalline structures (for example, when regular semicrystalline polymers are isothermally crystallized at temperatures close to *T*_*m*_). However, this is clearly not the case for the semiconducting polymers investigated here. Exemplary high-resolution transmission electron microscopy (HR-TEM) for the (spun-cast) D18 film shown in Figure 4a (and Figure S14 of the Supporting Information) evidence that the whole surface/volume of the material is underpinned by a dense arrangement of very small crystallites. Figure 4a shows a grain (orientation) map obtained using the LiveFFTColourMap script by Gatan Inc., which computes a grid of fast Fourier transformations (FFTs) in small regions of an HR-TEM image and then displays the orientation of the brightest spot in a color-coded map. A quantitative analysis (as described in the experimental part of the Supporting Information) reveals that the average size of crystallites is 1200 nm^2^, which corresponds to a diameter of ∼40 nm if circular crystals are assumed, and the density of crystallites is in the range of 10^10^ cm^–2^. We note that AFM images included in Figure S14 of the Supporting Information also suggest the presence of a dense arrangement of small crystallites. We argue that such a high density of nanoscopic crystallites indicate a crystallization scenario where crystal nuclei develop almost instantaneously, hindering the propagation and growth of crystallites over long distances (indeed, similarly to the case of regular semicrystalline polymers crystallized at temperatures very far from *T*_*m*_ and close to *T*_g_). The extremely fast overall crystallization of these materials would agree with this interpretation.

**Figure 4:**
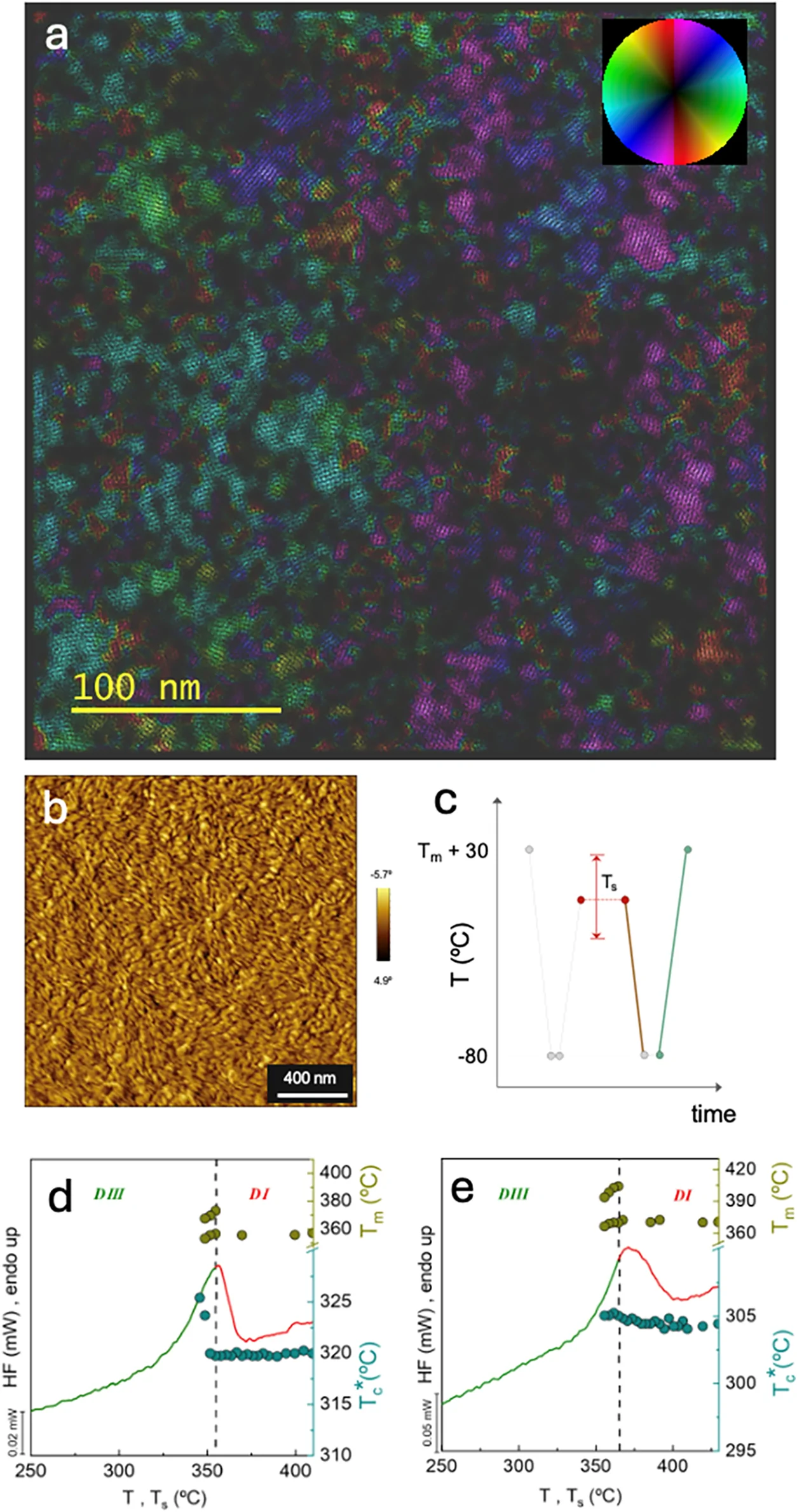
(a) HR-TEM micrograph of a spun-cast D18 film. The colored surface corresponds to material regions where (100) crystalline planes, thereby crystallites, are detected. Different colors represent the different crystal orientations, as shown in the color wheel. (b) Phase-contrast AFM image of a spun-cast D18 film. (c) Schematic representation of the FSC self-nucleation protocol. The blue line indicates the evaluated cooling trace and the green line indicates the evaluated heating trace. Results of the self-nucleation experiments for PTQ10 (d) and D18 (e). Solid lines are melting traces of crystals formed during cooling at −2000 °C/s and are shown as a reference to contextualize the results. Red lines and green lines indicate the temperature ranges for crystallization in Domain I and Domain III, respectively. The crystallization temperature during cooling, *T_c_* *, of crystals subjected to different *T*_s_s are shown as dark-green dots. The melting temperatures, *T*_*m*_s, of thus-formed crystals are shown as light-green dots.

We argue that this apparent discrepancy between the nucleation and growth rate constants and the observed solid-state nanomorphology might result from treating the crystallization process of these polymers in terms of a classical crystallization mechanism, which includes well-differentiated crystal nucleation and propagation steps. However, strictly, *k*_1_ + *k*_2_^′^ α^Φ^ ≈ *k*_2_^′^ α^Φ^ in our case (as *k*_2_^′^ ≫ *k*_1_), which could mean that crystallization in semiconducting polymers occurs through a single step. Thus, *k*_2_^′^ would not be the rate constant of the crystal growth but that of the formation of the small crystallites observed in HR-TEM and AFM. This process is likely more similar to a nucleation than a crystal growth process. Indeed, shown in Figure S15 of the Supporting Information is that the dependence of *k*_2_^′^ with *T_c_* follows a similar behavior as *n*, Φ, *T*_*m*_, and the ratio of (100) and (010) peak areas, especially for D18 (see Figure S16).

Motivated by the idea that crystallization of these polymers is governed by the formation of the crystallites, thereby by a process akin to nucleation, we performed self-nucleation experiments.^41,42^ More specifically, our aim was to figure out if the nucleation/crystallization of donor–acceptor semiconducting polymers can occur in the same three crystal nucleation domains observed in regular semicrystalline polymers. For that, the thermal protocol displayed in Figure 4c is applied in the FSC. Specifically, crystals formed by cooling from the melt at −2000 °C/s are taken to a given temperature *T*_*s*_ between the experimentally observed *T*_*m*_ and the equilibrium melting temperature, which is unknown for these polymers. After a given time at *T*_*s*_ (0.15 s in this case), samples are cooled and the crystallization temperature during cooling, T_c_*, is measured. Finally, the *T*_*m*_ of thus-formed crystals is probed in a subsequent heating scan. This protocol is repeated for different values of *T*_*s*_. For further details of the thermal protocol applied, see the experimental part of the Supporting Information.

Figure 4d,4e shows the results of the self-nucleation experiments for PTQ10 and D18. They display the dependence of *T*_*c*_ * with *T*_*s*_, from which the nucleation/crystallization domains are figured out, superimposed to the standard melting trace of crystals formed during cooling at −2000 °C/s. The cooling scans after the isothermal step at each *T*_*s*_ and the subsequent heating scans are included in Figure S16 of the Supporting Information. In regular semicrystalline polymers, the existence of surviving nuclei after partial melting at a given *T*_*s*_ leads to a self-nucleation Domain II, which is typically manifested as a progressive increase of the *T*_*c*_ * with decreasing *T*_*s*_.^42^ However, for both PTQ10 and D18, no such shift in the *T*_*c*_ * was observed when decreasing the *T*_*s*_ (see Figure 4d,4e), suggesting the absence of melt memory effects, i.e., aggregated polymer chains that trigger the crystallization in these materials. Instead, the systems exhibit a direct transition from Domain I, i.e., the isotropic melt domain, to Domain III, which is associated with annealing or recrystallization phenomena and thereby yields also a sharp increase of *T*_*c*_ * (see Figure 4d,4e). The absence of detectable Domain II suggests that crystallization in these semiconducting polymers is not promoted by classical point-like nuclei, the formation of which requires overcoming large energy barriers. In contrast, the formation of crystallites in these materials proceeds through the stack of adjacent polymer chains that require overcoming much lower energy barriers. Therefore, even if these polymers crystallize form isotropic, seed-free melts, the nucleation rate is extremely high. This interpretation is consistent with the above-mentioned difference between the values of *k*_2_^′^ and *k*_1_ and also with the solid-state nanomorphology found. Lastly, we would like to highlight that despite the formation of these crystallites seems to be almost instantaneous, their rate of formation (which is analogous to the primary nucleation and can be described as 1/*t*_*0*_) decreases as the *T*_c_ increases, as shown in Figure S17 of the Supporting Information.

## 3 Conclusions

In this work, we have introduced and validated a robust experimental methodology based on fast scanning calorimetry (FSC) that enables, for the first time, a reliable single-step determination of the ultrafast isothermal crystallization kinetics of high-performance, donor–acceptor semiconducting polymers, including D18 and PTQ10. A careful baseline correction allows us to overcome the intrinsic limitations of conventional DSC and the indirect “by-step” approaches, providing access to crystallization processes occurring within tenths of a second at high temperatures. Beyond the methodological advance, we demonstrate that the crystallization kinetics of these materials can be successfully described using a modified version of the Malkin model, also presented in this work. The new Malkin-based formalism, in principle, enables the separation of effective nucleation- and growth-related contributions and introduces a geometrical parameter that links kinetics with the crystal morphology. Our findings reveal a crystallization mechanism in semiconducting polymers that proceeds through a near-instantaneous formation of dense arrangement of nanoscopic crystallites, with kinetics effectively governed by a single dominant step rather than classical nucleation and growth processes. Because many modern conjugated polymers exhibit a similar solid-state morphology, we argue that this kinetic behavior and the resulting solid-state nanomorphology result from the high rigidity of backbones and a strong tendency of these semirigid molecules to aggregate via both π–π stacking and local lamellar-like self-assembly of backbones and side chains, which promotes an extremely fast nucleation rate. Moreover, we find two distinct crystallization kinetic behaviors (regimes) as a function of the crystallization temperature, which, as we demonstrate, are associated with the formation of crystals with different morphological characteristics. Together, these results redefine the understanding of crystallization in high-performance semiconducting polymers and provide a quantitative framework for correlating processing conditions, nanostructures, and device-relevant properties.

## Supplementary Material



## Data Availability

The datasets used to produce the figures in the article are included in the following https://doi.org/10.5281/zenodo.21719796.

## Notes

Experimental details, materials, and methods; detailed description of the modified Malkin model; motivation to investigate the crystallization of semiconducting polymers from the melt; procedure to extract the relevant heat flow rate signal during isothermal crystallization; comparison between direct isothermal crystallization analysis and stepwise crystallization strategies; Fourier transform infrared spectroscopy (FTIR) data for the polymers analyzed; isothermal crystallization kinetics of PEO; FSC heating traces for PTQ10 and D18 before and after the isothermal crystallization analysis; isothermal crystallization data for D18; isothermal crystallization experiments for PTQ10 and D18 samples with different thicknesses/sizes; fitting parameter Φ for different polymeric materials; crystallization exotherms and evolution of the crystalline fractions for PEO, PCL, P6, and P3HT crystallized at different crystallization temperatures; POM images for different polymeric materials; AFM images for D18 and PTQ10 supported onto Flash DSC chips; 2D WAXS detector images (in reciprocal space units) of PTQ10 and D18 isothermally crystallized on the Flash DSC chips; HR-TEM and AFM images of spin-coated polymer films; dependence of the *k*_2_´ rate constant with the crystallization temperature; FSC results of self-nucleation experiments; and dependence of the primary nucleation rate (as 1/*t*_0_) vs *T*_c_ (PDF)
