# Supplementary material for: Understanding the Ultrafast Crystallization Kinetics of Donor–Acceptor Semiconducting Polymers
Source: J Am Chem Soc. 2026 Aug 11;148(33):35694–704. doi: 10.1021/jacs.6c07715 (PMC13523684; doi:10.1021/jacs.6c07715)
Supplement: Supplementary file 1 [file ja6c07715_si_001.pdf]

Supporting Information for

Understanding the Ultrafast Crystallization Kinetics of Donor-Acceptor Semiconducting Polymers

Josep Tent-Pérez[1], Francisco Blanco-Vázquez[1], Jorge L. Olmedo-Martínez[1], Alberto Peinador Veiga[1], Matteo Sanviti[1], Marián Prada-Cortés[1], Antoine Curé[2], Eugenio Solla[3], Bernat Mundet[4], Cyril Aumaître[2], Xabier Martinez-Rodriguez[1], Renaud Demadrille[2], Jaime Martin[1,5] *

[1] Universidade da Coruña, Campus Industrial de Ferrol, CITENI, Campus de Esteiro, 15403, Ferrol, Spain

[2] IRIG-SyMMES, Université Grenoble Alpes/CEA/CNRS/Grenoble INP, 38000 Grenoble, France

[3] Universidade da Coruña, Servicios de apoyo a la investigación (SAI), Campus de Elviña, s/n, 15071 A Coruña, Spain.

[4] Catalan Institute of Nanoscience and Nanotechnology (ICN2), Campus UAB, Bellaterra, Barcelona, 08193, Catalonia, Spain

[5] Oportunius Programme, Axencia Galega de Innovación (GAIN), Xunta de Galicia, C/ Rúa Airas Nunes s/n, 15702, Santiago de Compostela, Spain

E-mail: jaime.martin.perez@udc.es

Experimental Part

Materials

Poly[[6,7-difluoro[(2-hexyldecyl)oxy]─5,8-quinoxalinediyl]─2,5-thiophenediyl] (**PTQ10**) (M_n = 82 kg/mol and Đ = 2.5) was synthesized as following: In a reaction vial, a mixture of 5,8-dibromo-6,7-difluoro-2-((2-hexyldecyl)oxy)quinoxaline (200 mg, 0.35 mmol, 1 eq.), 2,5-bis(trimethylstannyl)thiophene (145 mg, 0.35 mmol, 1 eq.), Pd_2dba_3 (6.5 mg, 0.007 mmol, 2 mol%.) and P(*o*-tolyl)$_3$ (8.6 mg, 0.028, 8 mol%.) was diluted in degassed dry toluene (17.7 mL, 20 mM)[1]. The reaction mixture is then heated at 110 °C and stirred. After 10 min, the reaction mixture was precipitated in a large volume of methanol and filtered. The crude product was purified through a Soxhlet apparatus with methanol, acetone, dichloromethane and chloroform. The chloroform fraction was concentrated under reduced pressure and precipitated in methanol to yield PTQ10 as a dark purple solid (127 mg, 73 % yield).

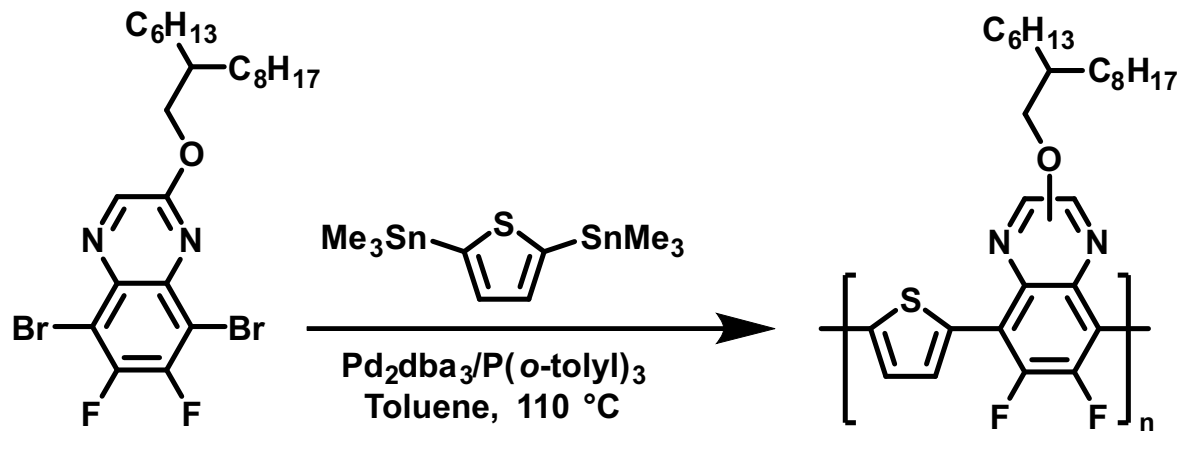

Poly[(2,6-(4,8-bis(5-(2-ethylhexyl)-4-fluoro)thiophen-2-yl)-benzo[1,2-b:4,5-b’]dithiophene)-alt-5,5'-(5,8-bis(4-(2-butyloctyl)thiophen-2-yl)dithieno[3',2':3,4;2",3":5,6]benzo[1,2-c][1,2,5]thiadiazole)] **(D18)** ($M_W = 63.4\ kg\ mol^{-1}$, Đ $= 1.9$) was purchased from Ossila.

Regioregular poly(3-hexylthiophene) (rrP3HT; $rr = 97.3\%$, $M_W = 74\ kg\ mol^{-1}$, Đ $= 2.1$) was also purchased from Ossila. Poly(ε-caprolactone) (PCL) ($M_W = 17.4\ kg\ mol^{-1}$, Đ $= 1.7$) and poly(ethylene oxide) (PEO) ($M_V = 100\ kg\ mol^{-1}$) were purchased from Sigma-Aldrich. Poly(1,6-hexanediol) (P6) with a $M_n = 3.5\ kg\ mol^{-1}$ was obtained following the procedure reported from Olmedo-Martínez et al.[2]

Samples investigated

All investigated samples are bulk, droplet-like pieces. Exemplary samples masses were e.g. 109 ng and 157 ng for PTQ10 and D18, respectively. These mass values are calculated from sample volumes (exemplary diameter and height values s were 100 µm and 22 µm, respectively, for PTQ10 sample, and were 95 µm and 33 µm, respectively, for D18) and considering a density of 1.1 g/mL).

Methods

Fast Scanning Calorimetry. Crystallization studies were performed on bulk samples (with an approximate mass around 100 ng) using a Flash DSC 1 made by Mettler Toledo. The temperature was controlled with an intercooler (TC100MT, Huber) with a range of working temperatures between -90 and 450 ºC. All ramps were performed with nitrogen as a purge gas with a constant

flow of 50 mL min^{-1}. Prior to utilization, chip sensors MultiSTAR UFS2 were conditioned and corrected according to Mettler Toledo recommendations.

In isothermal crystallization experiments of PTQ10, samples were first heated from 25 to 400 ºC at 1000 K s^{-1}, followed by an isotherm of 0.01 seconds at 400 ºC. Samples were then cooled down at 2000 K s^{-}1 to the desired isothermal crystallization temperature, T_c . Isothermal steps of 0.1-0.3 s were performed at each T_c to allow crystallization to saturate. Finally, the melting of crystals formed during the isothermal step was registered during a heating step from T_c to 400 ºC at 1000 K s^{-1}. For the analysis of D18 samples, a maximum heating temperature of 430 ºC was used. The thermal profile applied is shown in Figure 1

Self-nucleation experiments were conducted with the following procedure: (1) samples were heated 30 ºC above the melting temperature $(T_m + 30)$ ºC to erase thermal history (T_m is considered the melting peak temperature). (2) temperature was held for 0.15 s to allow the melting of existent thermally induced nuclei. (3) rapid cooling to a low temperature (-80 ºC) followed by a heating step to a specific temperature denoted T_s and held for 0.15s. (4) After a 0.15 s at T_s samples were cooled again to -80 ºC and then heated to $(T_m + 30)$ ºC. All heating and cooling ramps were performed at a rate of 2000 ºC s^{-1}. The analysed T_s ranged from 430 ºC to 344 ºC and 350 ºC for PTQ10 and D18, respectively ºC

Differential Scanning Calorimetry. A DSC25 (TA Instruments) equipped with an intercooler was used. Indium and Tin standards were used to calibrate the equipment. All experiments were conducted under nitrogen atmosphere at a constant flow of 50 mL min^{-1}. Isothermal crystallization experiments were conducted on polyethylene oxide (PEO), polycaprolactone (PCL), poly(1,6-hexanediol) (P6), and poly (3-hexylthiophene) (P3HT) samples of ~5 mg. Samples were first heated well above their T_m and held for 3 min to erase any previous thermal history. Then, samples were cooled to the desired T_c and held at until complete crystallization. All measurements were conducted in aluminum pans with non-hermetic lids, at scanning rates of 20 ºC min^{-1} for both cooling and heating traces.

Wide Angle X-ray Scattering. Transmission WAXS measurements were conducted on polymer samples deposited in MultiSTAR UFS2 chip sensors at the BL11 NCD-SWEET beamline at the ALBA Synchrotron Radiation Facility in Spain. The incident beam energy was set to 12.4 keV, with a channel-cut Si (111) monochromator, collimated with an array of Be Compound Refractive Lenses. The scattering patterns were recorded using a Rayonix LX255-HS area detector, with a pixel array of 1920 × 5760 pixels (H × V) and a pixel size of 44.54 × 44.54 μm^2 with a binning mode of 2 × 2. The scattering vector, q, was calibrated using Cr_2O_3 as a standard sample (NIST), with a sample-to-detector distance of 202.37 mm. Integration scan of 1 s was applied.

Polarized Light Microscopy. The identification of the different crystal morphologies was carried out with a polarized light optical microscope (Olympus BX51), equipped with an Olympus SC50 digital camera with a Linkam-15 tp-91 hot stage and coupled to a liquid nitrogen cooling system.

Atomic Force Microscopy Images. Atomic force microscopy (AFM) images were acquired using a CSInstruments Nano-Observer AFM, operated in resonant mode in air at room temperature. AppNano ACT silicon cantilevers with no coating on the detector side and nominal resonance frequency of 300 kHz were used. The images were recorded with scan sizes of 3 μm^2, resolutions of 512×512 pixels, and scan speeds of 0.75 and 1 line per second, adjusting the amplitude setpoint to minimise lateral forces without loss of resolution. Image processing (such as plane levelling and line correction) was performed using Gwyddion v2.66 software.

High-Resolution Electron Microscopy (HR-TEM). Low-dose aberration-corrected TEM images were acquired in a double-corrected (S)TEM Thermofisher Spectra 300 operated at 300 KV. A direct detection K3 camara was used for the acquisition installed in a Continuum Energy-filter spectrometer from GATAN. Dose rates of around 1-3 e^-/pixel/s were used to minimize sample damage. The grain orientation maps were obtained applying the LiveFFTColourMap script by Ben Miller (Gatan Inc.) which computes a grid of fast Fourier Transformations (FFTs) in small regions of an HRTEM image and then displays the orientation (tetha angle) of the brightest spot in a color-coded map [Miller, B. (2022). *Live FFT color map* [DigitalMicrograph Script]. Gatan, Inc. Scripts Library. https://www.gatan.com/resources/scripts-library]. We developed an additional custom Python script to calculate the crystallinity percentage and average crystal size, from the grain orientation maps data.

Description of the modified version of the Malkin model

First, we need to present the general macrokinetic equation from which all the development will be done:

$$\dot{\alpha} = \dot{\alpha}(T, \alpha) \tag{1}$$

where $\dot{\alpha}$ is the rate of crystallization, α is the degree of crystallinity varying from 0 to the equilibrium value (generally considered to be 1).

In order to get a full description of the crystallization process, both nucleation and crystal growth phenomena must be considered in the equation (eq.1). In this way, $\dot{\alpha}$ is composed by two stages:

$$\dot{\alpha}(T, \alpha) = \dot{\alpha}_1(T, \alpha) + \dot{\alpha}_2(T, \alpha) \tag{2}$$

The function $\dot{\alpha}_1$ describes the nucleation and $\dot{\alpha}_2$ represents the crystal growth. Same approach used for the determination of the macrokinetics of chemical reactions is taken to describe the form of $\dot{\alpha}_1$ and $\dot{\alpha}_2$. Therefore, a function of two variables will be represented as the product of two functions each of which depends only on one variable:

$$\begin{aligned} \dot{\alpha}_1(T, \alpha) &= f_1(T) f_2(\alpha) \\ \dot{\alpha}_2(T, \alpha) &= f_3(T) f_4(\alpha) \end{aligned} \tag{3}$$

Nucleation phenomenon is governed by the rate of formation of primary nuclei $I(T)$, and the volume of the critical equilibrium nucleus $V_*(T)$. The rate of formation of nuclei can be expressed as:

$$I(T) = I_0 \exp\left(-\frac{E}{RT} - \frac{\psi_1 (T_m^0)^m}{T(T_m^0 - T)^m}\right) \tag{4}$$

being I the number of nuclei formed per unit time per unit volume; I_0, ψ_1 and m constants; E representing the activation energy of transfer of a polymeric segment through the nucleus-melt interface; R is the gas constant; and T_m^0 the equilibrium melt temperature.

Now we introduce the first change respect to the Malkin model. In the paper, the author suggests considering the kinetics of nuclei formation as an analogue to a first order chemical reaction. However, this simplification might lead to a deviation of the model from the experimental data. Thus, an n_M order chemical reaction will serve as the analogue to the nuclei formation rate:

$$f_2(\alpha) = \left(\alpha_{eq} - \alpha\right)^{n_M} \tag{5}$$

As mentioned before, the degree of crystallinity varies from 0 to the equilibrium value represented by α_{eq}. For our case study, the experimental data is normalized to (0,1) and α_{eq} will then be considered as 1, in order to match up the model with the experimental data.

At this point, the evolution of the crystallization rate associated to primary crystallization is represented on the following form:

$$\dot{\alpha}_1(T,\alpha) = V_*(T)\, I_0 \exp\left(-\frac{E}{RT} - \frac{\psi_1 (T_m^0)^m}{T(T_m^0 - T)^m}\right)(1-\alpha)^{n_M} \tag{6}$$

Our objective is to get a model that represents the isothermal crystallization observed on a semiconductive polymer (PTQ10). If the temperature remains constant, the equation 6 can then be written as:

$$\dot{\alpha}_1(\alpha) = k_1 (1-\alpha)^{n_M} \tag{7}$$

where $k_1 = V_*(T)\, I_0 \exp\left(-\frac{E}{RT} - \frac{\psi_1 (T_m^0)^m}{T(T_m^0 - T)^m}\right)$.

Following the description of the evolution of crystallization rate, the second stage known as crystal growth is governed by the generalized equation of the growth rate:

$$G(T) = G_0 \exp\left(-\frac{E}{RT} - \frac{\psi_2 (T_m^0)^l}{T(T_m^0 - T)^l}\right) \tag{8}$$

where G_0, ψ_2 and l are constants. In the same form as for the function $f_2(\alpha)$, the crystal growth rate will be analogue to an n order kinetic equation:

$$f_4(\alpha) = \left(\alpha_{eq} - \alpha\right)^{n_M} \tag{9}$$

Now the equation for $\dot{\alpha}_2(\alpha, T)$ is described as:

$$\dot{\alpha}_2(T,\alpha) = G_0 \exp\left(-\frac{E}{RT} - \frac{\psi_2 (T_m^0)^l}{T(T_m^0 - T)^l}\right) S(\alpha)(1-\alpha)^{n_M} \tag{10}$$

Following the same consideration stablished for $\dot{\alpha}_1(\alpha, T)$, if temperature is kept constant the equation can be rewritten:

$$\dot{\alpha}_2(\alpha) = k_2\, S(\alpha)(1-\alpha)^{n_M} \tag{11}$$

where $k_2 = G_0 \exp\left(-\frac{E}{RT} - \frac{\psi_2 (T_m^0)^l}{T\left(T_m^0 - T\right)^l}\right)$, and $S(\alpha)$ is a function that describes the total surface area over which crystal formation occurs.

If we substitute $\dot{\alpha}_1(\alpha)$ and $\dot{\alpha}_2(\alpha)$ into equation (2), the general macrokinetic equation for polymer isothermal crystallization is obtained by:

$$\frac{d\alpha}{dt} = (k_1 + k_2\, S(\alpha))(1-\alpha)^{n_M} \tag{11}$$

Considering the geometry of the crystals formed by the studied polymer, the form of the function $S(\alpha)$ could be assessed. In the paper, Malkin takes the linear form of $S(\alpha)$ as $S(\alpha) = D\alpha$ to simplify equation 11 and get an analytical solution. Contrary, we will consider that the surface area over crystal formation occurs has the from $S(\alpha) = D\alpha^{\Phi}$ as the general nature of the function is considered to be non-linear. Finally, the isothermal crystallization model applied to fit the experimental data is defined as:

$$\frac{d\alpha}{dt} = \left(k_1 + k_2'\, \alpha^{\Phi}\right)(1-\alpha)^{n_M} \tag{11}$$

where $k_2' = k_2 D$ and Φ is a constant that captures how the surface area involved in crystal formation scales with the volume (or fraction) of material that has already transformed. Therefore, Φ gives information about the morphology of the growing phase and the dimensionality of growth:

**Φ** value	
$\mathbf{\Phi = 0}$	Surface area constant during crystallization
$\mathbf{\Phi = 1}$	Surface area grows linearly with transformed fraction
$\mathbf{\Phi > 1}$	Surface area grows faster than transformation e.g., dendritic or fractal-like growth
$\mathbf{0 < \Phi < 1}$	Surface area grows slower than transformation e.g., compact or layered growth

General Interpretation of the Avrami index, n:

The Avrami index can be expressed as the sum of two terms: one is related to the dimensionality of the growing crystals (n_d), and the other represents the contribution of the time dependence of the nucleation (n_n)[3] :

$$n = n_d + n_n$$

where n_d values can only be integer numbers going from 1 to 3, representing the dimensionality of the crystals formed. In the case of polymeric compounds, n_d values of 2 or 3 are signature of axialtic and spherulitic growth, respectively. On the other hand, n_n values are comprised between 0 and 1, with $n_n = 0$ representing an instantaneous nucleation, and $n_n = 1$ corresponding to sporadic nucleation[4,5].

Motivation to investigate the crystallization kinetics of semiconducting polymers from the melt.

A first motivation to investigate the crystallization kinetics of semiconducting polymers from the melt at such temperatures is that, to our knowledge, there is not a suitable kinetic model proving mechanistic information for the crystallizations of polymers from solutions in which the solvent evaporates. Therefore, we argue that crystallization from the melt is nowadays a reasonable first approach to investigate how the crystallization of (semi)rigid semiconducting polymer occurs.

Moreover, it is known that crystallization from the melt and from solutions (induced by solvent evaporation) share similar crystallization pathways. In both cases, the crystallizable polymer liquid is a dense –often entangled—ensemble of polymer chains with no possibility for large-scale center-of-mass diffusion in the time scales in which the crystallization process occurs. Therefore, as opposed to e.g. crystallization of supercooled solutions (where concentration is kept constant and temperature is decreased), the crystallizing polymer chains cannot diffuse to crystal growth fronts and propagate the crystals. Instead, crystallization occurs rather “locally”. As a result, in flexible semicrystalline polymers, both crystallization from the melt and from solutions (induced by solvent evaporation) frequently result in similar spherulitic morphologies (here again as opposed to e.g. crystallization of supercooled solutions, which can result in the formation of large, independent crystallites). Semiconducting polymer films rarely develop spherulites but, as shown in the Figure S1a, both melt crystallization and solution crystallization (specifically spin coating) result in very similar –almost indistinguishable—solid-state morphologies. We must note that poly(3-hexyltiophene) (P3HT) has been analyzed here because it has a lower Tm and therefore allows being crystallized from the melt without significant degradation.

Interestingly, the similarities between melt and solution crystallization kinetics can be also inferred from isothermal crystallization curves. Shown below are the crystallization kinetic curves obtained by in-situ GIWAXS during blade coating of PTQ10 films. As can be clearly observed in Figure S1b, the crystallization curves have also sigmoidal shape as those of crystallization from the melt.

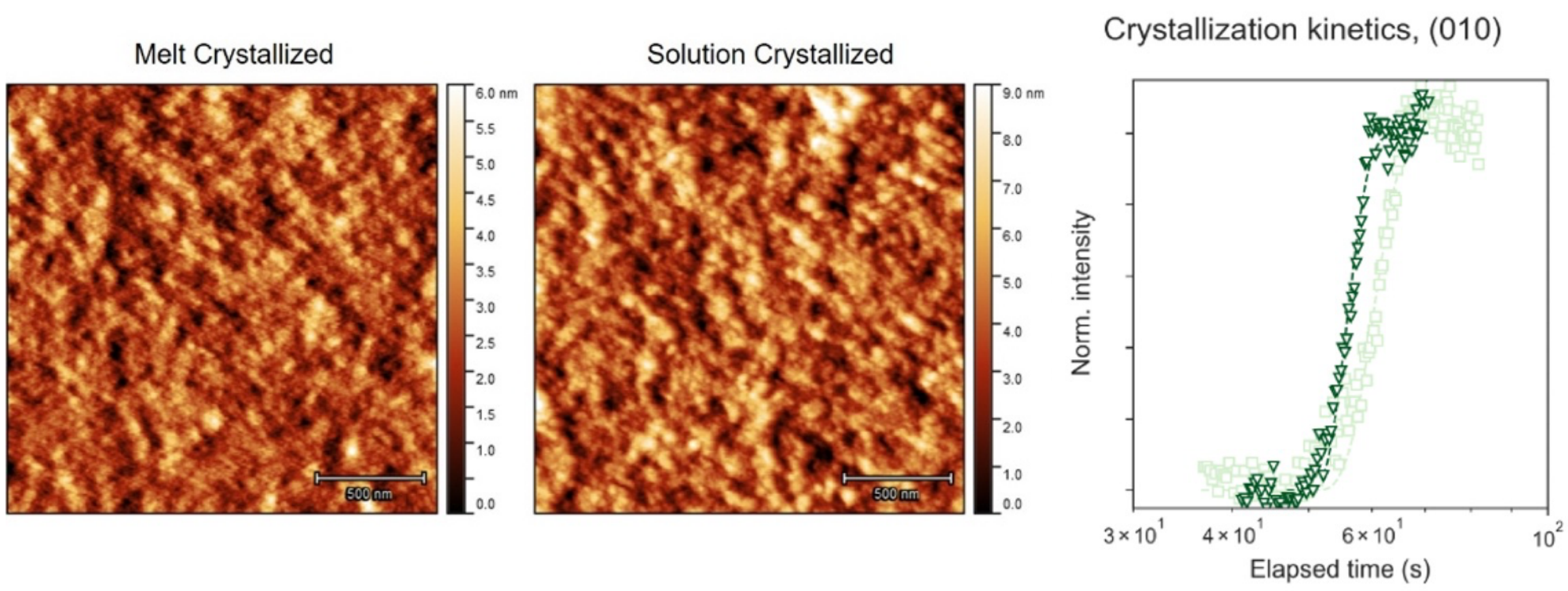

Figure S1. (a) Solid-state morphologies of melt crystallized, and solution crystallized (spin coated) P3HT films. (b) In situ GIWAXS data during the formation of a PTQ10 film by blade coating. Dark and light green symbols show evolution of the intensity of the (010) peak over time for films cast at 2 different temperatures.

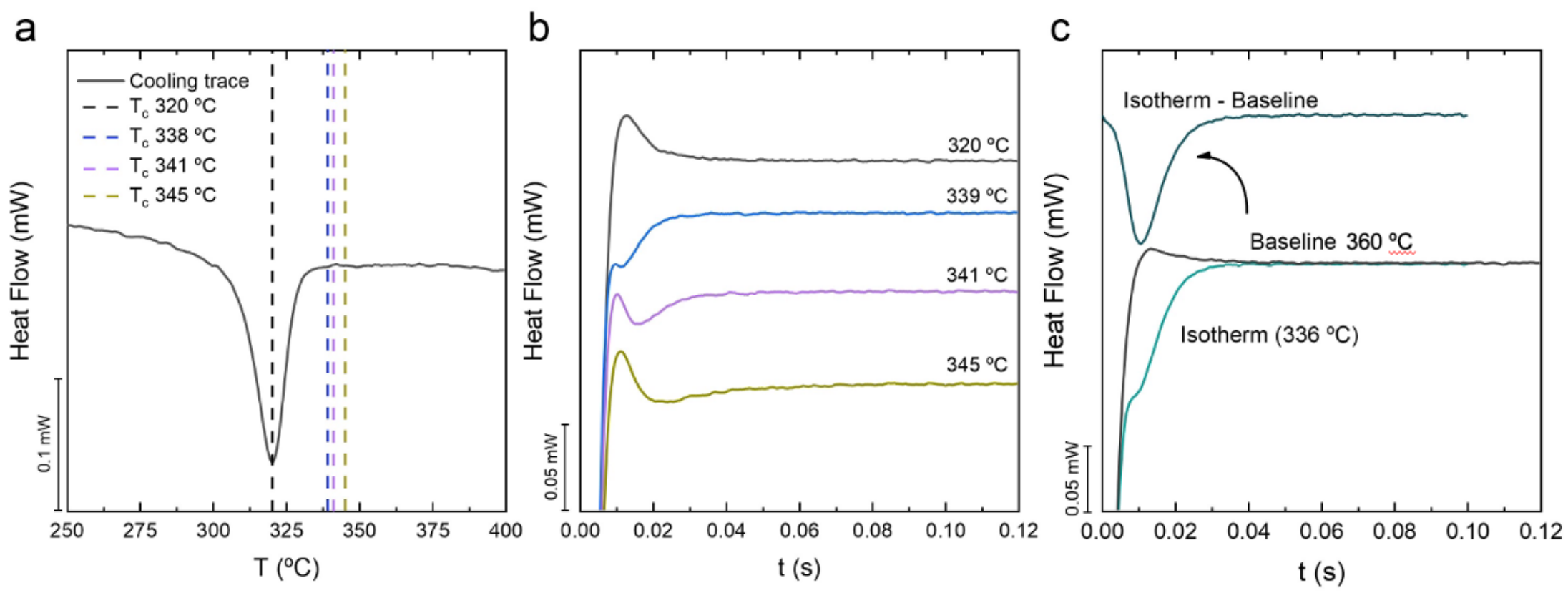

Figure S2. **Extraction of the crystallization exotherms from Flash DSC isothermal experiments.** (a) Identification of different crystallization temperatures (T_c) on the cooling trace of PTQ10 bulk sample. (b) Raw HF signal during isothermal crystallizations at the indicated temperatures. (c) Baseline subtraction on an isothermal HF raw trace. All thermograms were represented with endothermic events upside.

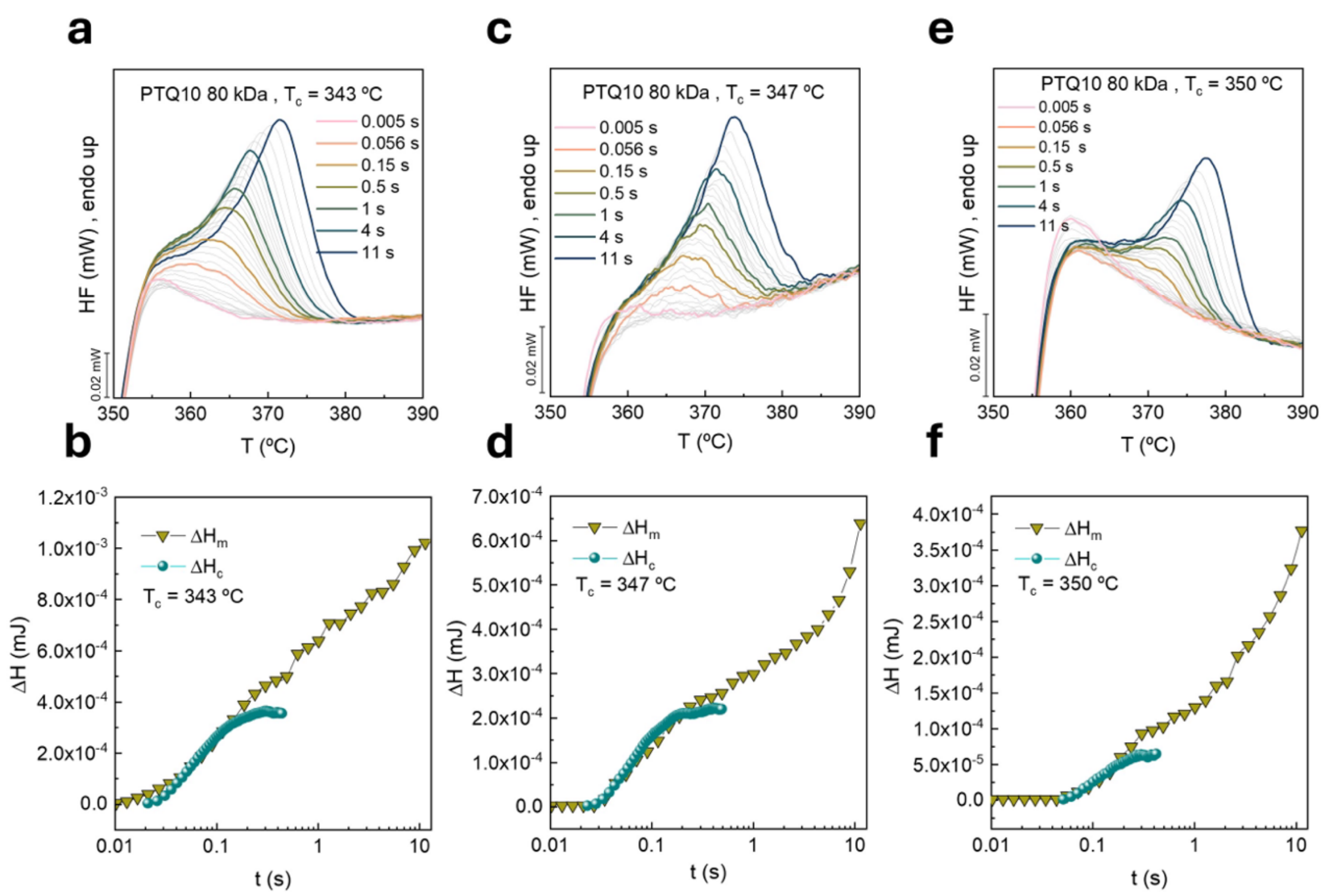

Figure S3. Comparison between direct isothermal crystallization and stepwise crystallization experiments for PTQ10. (a, b, c) Crystal melting evolution for different isothermal times of samples crystallized at 343, 347 and 350 ºC, respectively; **(d, e, f)** comparison between melting enthalpy (ΔH_m) extracted from the "by steps" method and crystallization enthalpy (ΔH_c) extracted from the "direct method" for PTQ10 samples isothermally crystallized at 343, 347 and 350 ºC, respectively. All thermograms were represented with endothermic events upside.

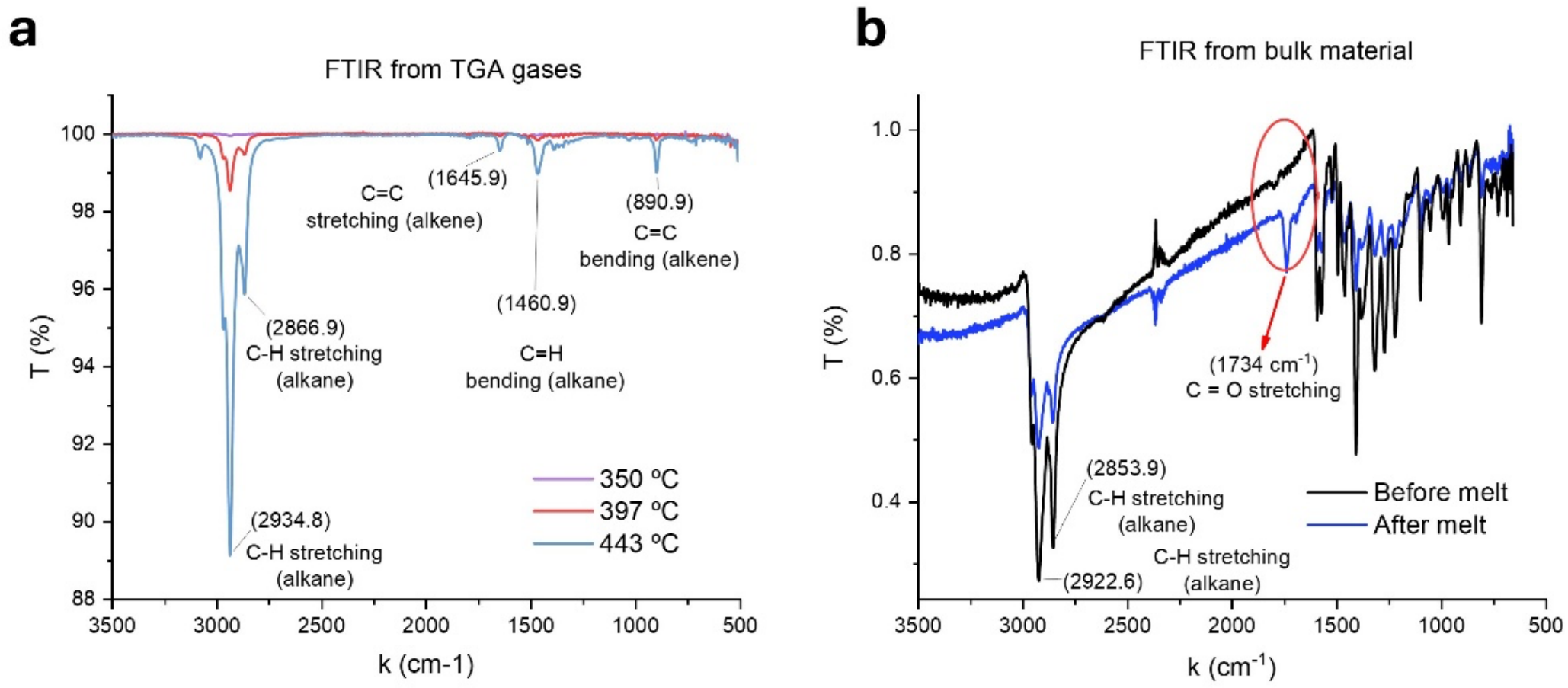

Figure S4. Fourier Transformed Infrared spectroscopy (FTIR) of PTQ10 performed on: outcoming gases from thermogravimetric measurements at 350 ºC, 397 ºC and 443 ºC (a), bulk material before and after being melted (heated up to 400 ºC) (b).

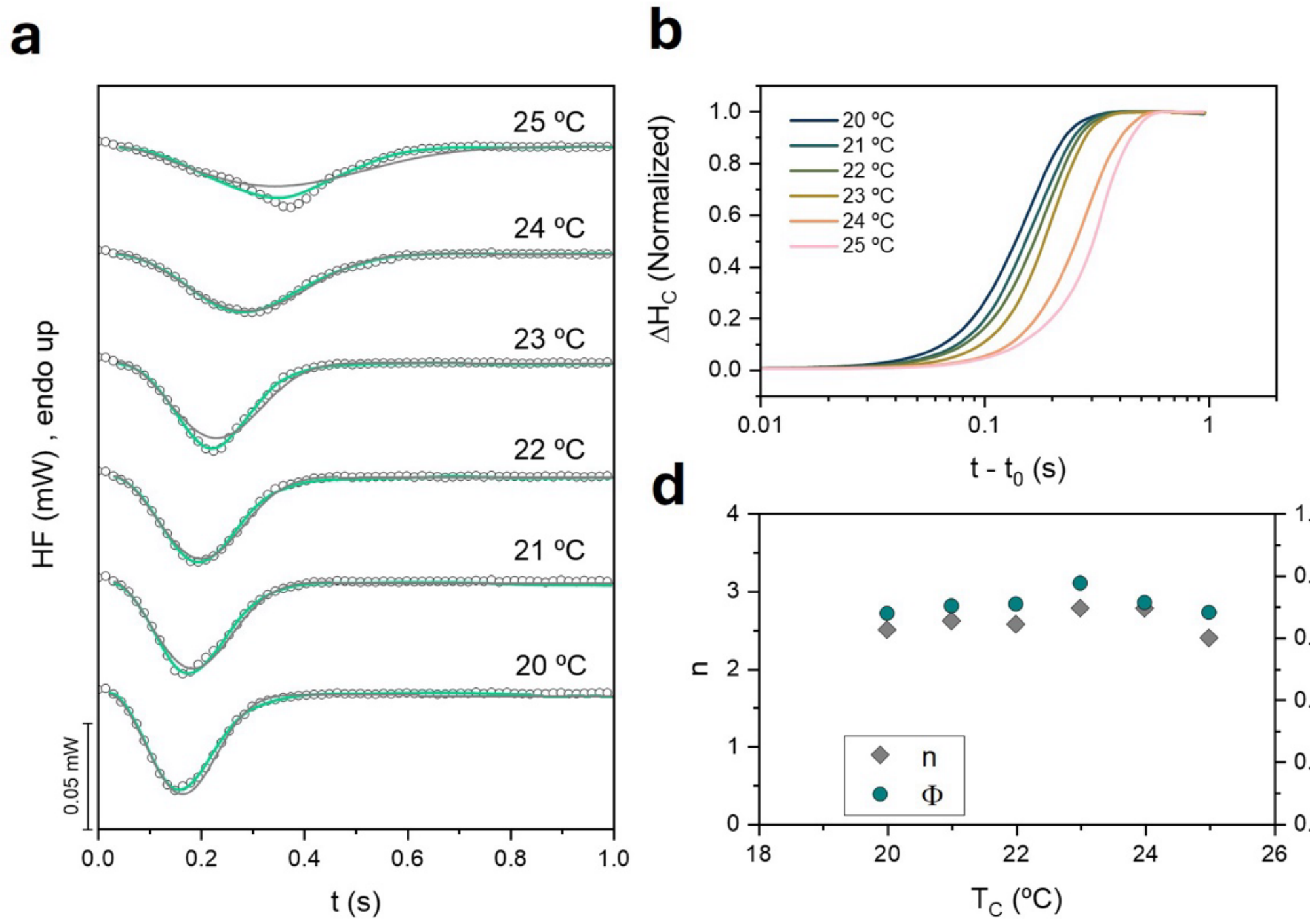

Figure S5 | (a) Isothermal crystallization exotherms of PEO at indicated T_cs. Hollow circles are experimental data, grey lines are fits to Avrami model and green lines are fits to Malkin model. **(b)** Normalized advance of crystallization curves at each isothermal temperature. **(c)** Evolution of the Avrami exponent (n) and Malkin parameter (Φ) expressed as a function of T_cs **(d)**.

Table S1. Fitting parameters of the Avrami and Malkin models for isothermally crystallized PEO

	PEO							
	Avrami			Malkin				
T (ºC)	k (s^{-n})	n	R^2	k1 (s^{-1})	k2' (s^{-1})	Φ	n_M	R^2
**20**	128.7	2.71	0.99993	0.014	18.6	0.68	0.91	0.99992
**21**	81.3	2.60	0.9996	0.048	18.1	0.71	0.93	0.9998
**22**	72.3	2.67	0.99991	0.038	16.2	0.71	0.86	0.9999
**23**	64.0	2.77	0.9998	0.059	17.7	0.78	0.90	0.99995
**24**	27.6	2.75	0.9994	0.030	11.1	0.72	0.88	0.99998
**25**	10.0	2.38	0.9996	0.086	9.8	0.79	0.80	0.9995

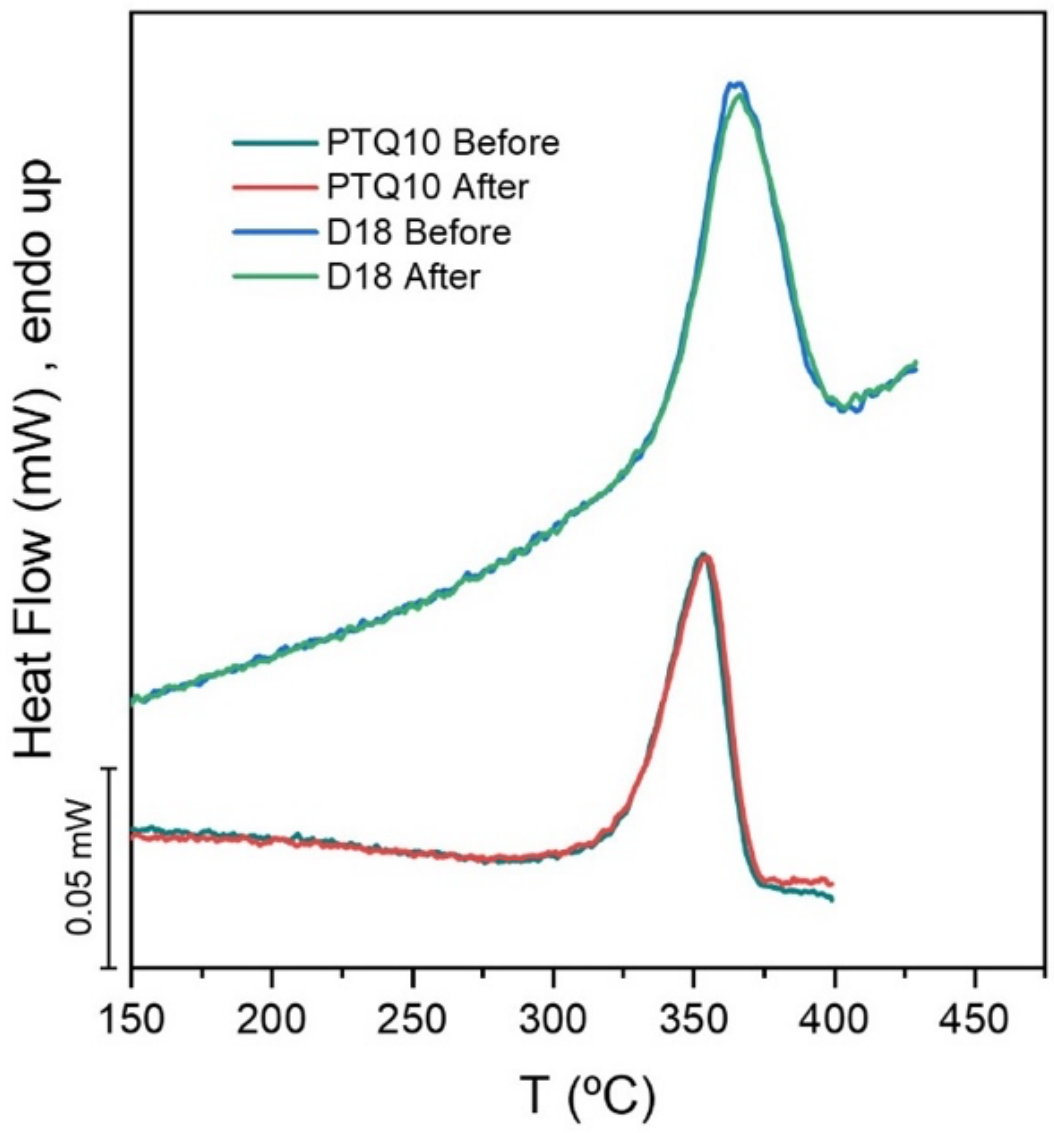

Figure S6 Heating traces of PTQ10 and D18 (at 2000 ºC/s, after being cooled at 2000 ºC/s) before and after the FSC isothermal experiments.

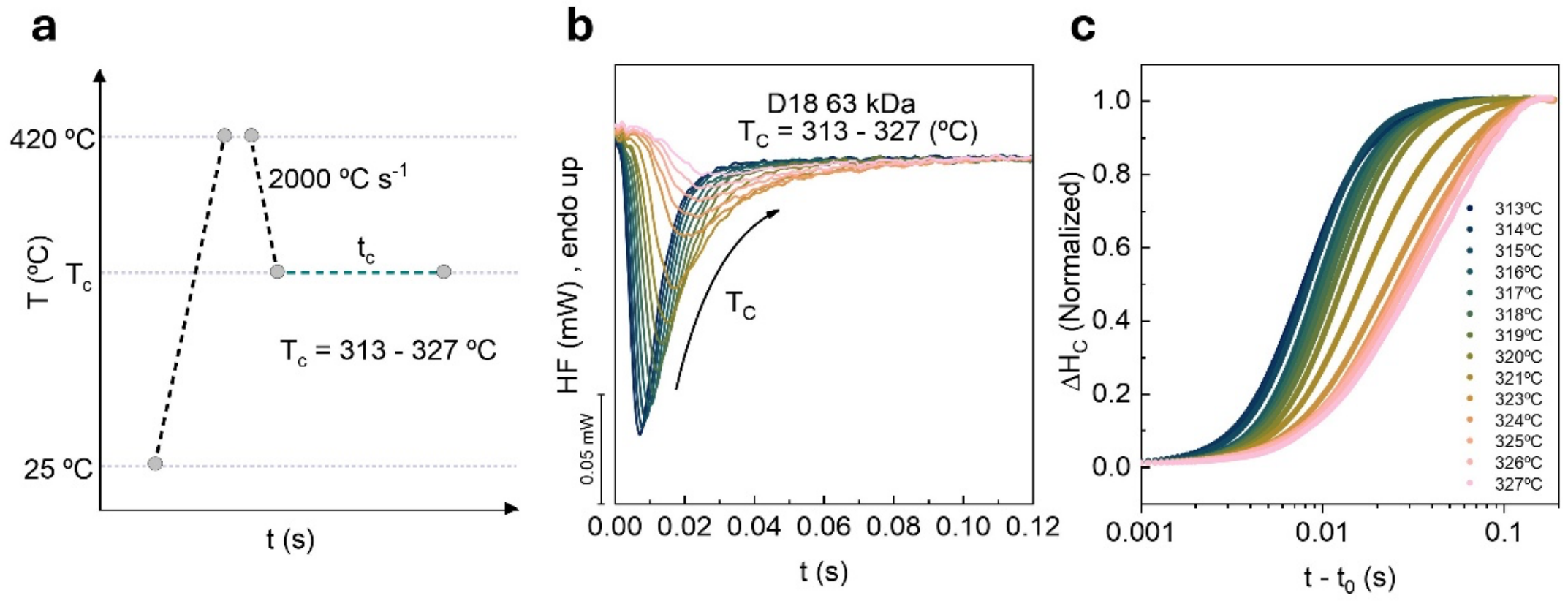

Figure S7 Flash DSC isothermal crystallization experiments for D18 sample cooled from melt state: (a) Isothermal crystallization protocol applied in the FSC; **(b)** crystallization exotherms for D18 crystallized at different crystallization temperatures (from 313 to 327ºC) (thermograms are represented with endothermic events upside.; **(c)** Evolution of the crystalline fraction with crystallization time for the different crystallization temperatures.

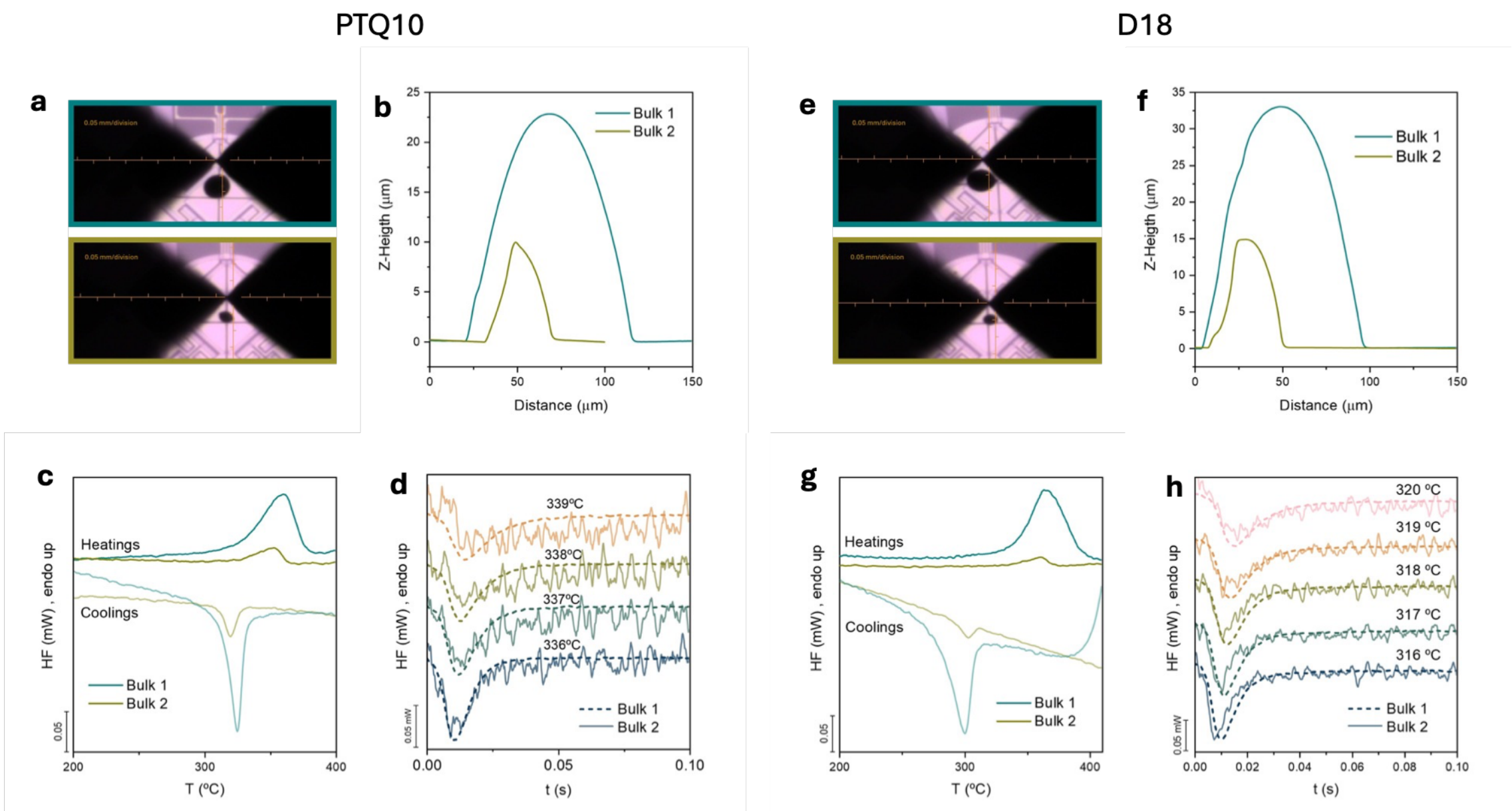

Figure S8 Flash DSC isothermal crystallization experiments for PTQ10 and D18 samples with different thickness/size: (a, e) Optical photographs of large (denoted as bulk 1) and small (denoted as bulk 2) samples (black circles) while being measured by mechanical profilometry. **(b, f)** Height profiles of the measured large (bulk 1) and small (bulk 2) samples. **(c,g)** Melting and cooling curves from FSC. **(d,h)** HF curves recorded for large (bulk 1) and small (bulk 2) samples isothermically crystallized at indicated crystallization temperatures.

Table S2. Standard error (S) and coefficient of determination (R^2) obtained from the Avrami and Malkin fits performed on PTQ10 and D18 isothermal crystallization curves.

	PTQ10							
	Avrami			Malkin				
T (ºC)	S_k	S_n	R^2	S_{k1}	$S_{k2'}$	S_{Φ}	S_{nM}	R^2
**336**	3.96E+04	1.82E-02	0.9990	1.68E-04	1.44	1.92E-03	5.18E-03	0.9998
**337**	2.45E+04	7.38E-03	0.9998	1.16E-05	0.87	9.18E-04	4.15E-03	0.99994
**338**	4.99E+04	1.80E-02	0.9991	3.46E-06	1.04	8.92E-04	6.02E-03	0.9998
**339**	1.39E+04	7.50E-03	0.9997	2.56E-09	0.38	4.30E-04	2.49E-03	0.9998
**340**	4.39E+03	2.90E-03	0.99997	6.03E-05	0.72	1.05E-03	4.83E-03	0.997
**341**	6.14E+03	1.04E-02	0.9993	6.19E-09	0.18	1.13E-04	4.09E-03	0.998
**342**	4.51E+02	7.14E-03	0.9995	4.15E-06	0.54	1.20E-03	5.66E-03	0.993
**343**	3.58E+01	5.41E-03	0.9994	8.86E-08	0.23	8.13E-04	3.79E-03	0.995
**344**	1.41E+01	4.11E-03	0.9995	3.56E-04	0.39	2.21E-03	8.02E-03	0.98
**345**	1.18E+01	5.75E-03	0.9990	2.68E-08	0.10	3.24E-04	4.40E-03	0.990
	**D18**							
	**Avrami**			**Malkin**				
**T (ºC)**	S_k	S_n	R^2	S_{k1}	$S_{k2'}$	S_{Φ}	S_{nM}	R^2
**313**	2.42E+03	5.02E-03	0.99991	3.28E-07	0.24	1.86E-04	8.09E-04	0.9998
**314**	6.60E+02	2.46E-03	0.99998	2.25E-06	0.51	4.39E-04	2.13E-03	0.9998
**315**	1.09E+03	2.56E-03	0.99997	1.28E-07	0.39	5.10E-04	1.36E-03	0.9995
**316**	2.33E+03	3.45E-03	0.99995	3.64E-06	0.38	3.10E-04	1.65E-03	0.99993
**317**	3.31E+03	6.96E-03	0.9998	1.30E-09	0.20	2.34E-04	8.07E-04	0.9998
**318**	6.42E+02	2.91E-03	0.99996	2.94E-09	0.33	5.43E-04	1.45E-03	0.99991
**319**	4.03E+02	3.11E-03	0.99996	1.28E-06	0.20	2.81E-04	1.00E-03	0.9998
**320**	8.33E+01	9.61E-04	0.99999	1.84E-07	0.31	4.56E-04	2.05E-03	0.9995
**321**	9.98E+01	3.55E-03	0.9998	2.21E-08	0.42	1.23E-03	3.54E-03	0.998
**323**	4.17E+01	5.43E-03	0.9995	1.74E-08	0.33	1.12E-03	5.28E-03	0.992
**324**	2.20E+01	5.29E-03	0.9994	3.59E-05	0.48	2.22E-03	8.15E-03	0.990
**325**	1.88E+01	6.36E-03	0.9990	9.01E-08	0.34	1.78E-03	6.83E-03	0.990
**326**	1.37E+01	6.05E-03	0.9990	2.77E-08	0.28	1.69E-03	6.34E-03	0.994
**327**	9.01E+00	6.19E-03	0.998	9.70E-07	0.45	2.86E-03	1.04E-02	0.98

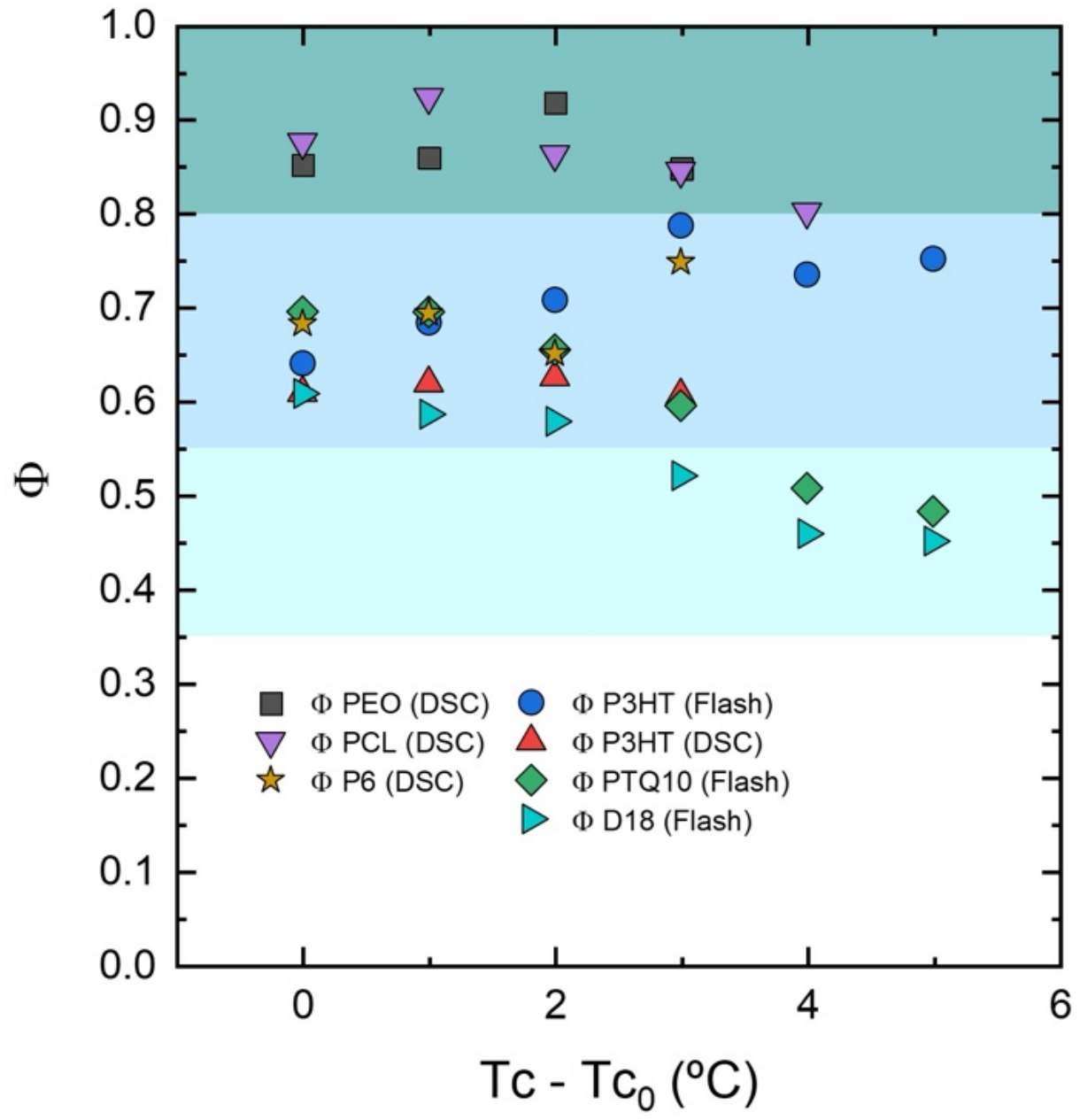

Figure S9. Fitting parameter *Φ* for different polymeric materials. Evolution of the Malkin (Φ) parameter of PEO, PCL, P6, P3HT, PTQ10 and D18 samples at different crystallization temperatures (T_c). Results are expressed as a function of the difference between the T_c and the lowest evaluated temperature (T_{c_0}). Thermal treatments were carried out with conventional DSC, except to P3HT, which was treated with both DSC and FSC in order to compare the analysis from both techniques

The extended Malkin model was also tested with crystallization curves of different polymeric materials: polyethylene oxide (PEO), polycaprolactone (PCL), poly(1,6-hexanediol) (P6), and poly (3-hexylthiophene) (P3HT). Thermal treatments were carried out with conventional DSC, except to P3HT, which was treated with both DSC and FSC in order to compare the analysis from both techniques.

To be able to compare different materials, Φ was represented as a function of the difference between the lowest evaluated crystallization temperature (T_{c_0}) and the represented crystallization temperature (T_c). Figure S5 shows the evolution of Φ values with the crystallization temperature (T_c), for the above mentioned polymers. Three different ranges of Φ values were identified. First, PEO and PCL presented the highest values of Φ. Both polymers are commonly classified with a three-dimensional crystal morphology due to their characteristic spherulitic growth[6,7]. Consecutively, P6 and P3HT presented lower values oscillating between 0.6 and 0.8. For P6, the formation of axialites has been reported under isothermal crystallization at 40 ºC[8]. In the case of P3HT, two different crystal forms (form I and form II) can be found depending if the side chains present interdigitation or not[9]. For example, Kuebler et al. reported the formation of spherulites when crystallizing thin films from melt state[10] However, P3HT crystal morphology also has been associated with crystalline nanofibrils when spin-coated from solution[9].

Given the observed correlation between Φ and the Avrami exponent n, a generalized correspondence between Φ values and the dimensionality of the crystals formed during isothermal crystallization was built. With a similar approach of what has been done in other works for n_D values, Table S6 shows the relationship that we found between Φ value and the corresponding crystal morphology. At high values of Φ (from 1 to 0.8), a preferential spherulitic growth was observed. Below 0.8 and above 0.6, structures with lamellar axialitic growth predominated. Then, Φ values between 0.6 and 0.4 are suggested to be 2D planar crystallites and Φ values below 0.4 could be related to the formation of 1D crystals. Except to PTQ10 and D18 polymers, which do not present birefringent structures, the identification of the different crystalline features present in the analysed polymers was further confirmed with polarized light optical microscopy technique (PLOM) (see Figure S11).

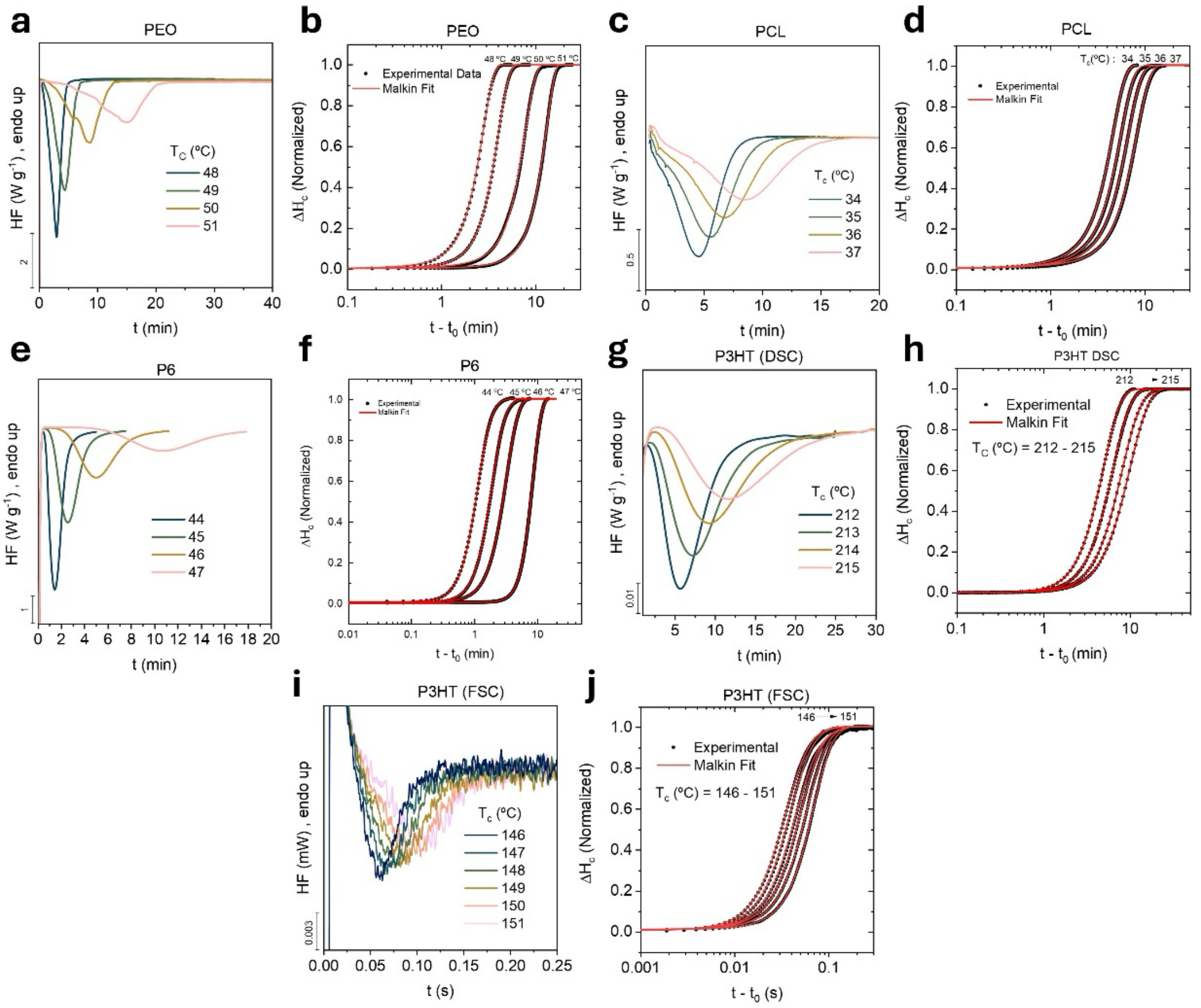

Figure S10. Crystallization exotherms and evolution of the crystalline fractions for PEO (a,b), PCL (c,d) P6 (e,f), P3HT (g,h,i,j) crystallized at different crystallization temperatures. Thermal treatments were carried out with conventional DSC, except to P3HT, which was treated with both DSC and FSC in order to compare the analysis from both techniques

Table S3. Malkin fit parameters for PEO and PCL.

	Malkin Parameters								
	PEO				PCL				
T (ºC)	*k1* *(min^{-1})*	*k2'* *(min^{-1})*	*Φ*	n_M	T (ºC)	*k1* *(min^{-1})*	*k2'* *(min^{-1})*	*Φ*	n_M
**48**	0.0170	1.29	0.85	0.68	**34**	0.0192	0.63	0.95	0.80
**49**	0.0078	0.97	0.86	0.72	**35**	0.0162	0.58	0.88	0.75
**50**	0.0072	0.51	0.92	0.71	**36**	0.0160	0.51	0.92	0.79
**51**	0.0020	0.21	0.85	0.57	**37**	0.0095	0.40	0.86	0.79

Table S4. Malkin fit parameters for P6 and P3HT (DSC).

	Malkin Parameters								
	P6				P3HT (DSC)				
T (ºC)	*k1* *(min^{-1})*	*k2'* *(min^{-1})*	*Φ*	n_M	T (ºC)	*k1* *(min^{-1})*	*k2'* *(min^{-1})*	*Φ*	n_M
**44**	0.0028	2.64	0.68	1.48	212	3.52E-4	0.52	0.61	1.00
**45**	0.0028	1.59	0.69	1.45	213	6.02E-4	0.41	0.62	1.00
**46**	0.0021	0.84	0.65	1.33	214	0.0011	0.32	0.63	1.00
**47**	1.24E-08	0.47	0.75	1.47	215	0.0011	0.25	0.61	1.00

Table S5. Malkin fit parameters for P3HT (FSC).

	Malkin Parameters			
	P3HT (FSC)			
T (ºC)	*k1* *(s^{-1})*	*k2'* *(s^{-1})*	*Φ*	n_M
**146**	0.714	71.81	0.64	1.09
**147**	0.913	68.03	0.68	1.07
**148**	0.699	64.38	0.71	1.20
**149**	1.308	59.96	0.79	1.12
**150**	0.815	48.44	0.74	1.03
**151**	0.594	43.63	0.75	0.99

Table S6. Relationship between *Φ* and crystal morphology.

**Φ** value	**Morphology**
$0.8 < \Phi < 1$	3D spherulitic growth
$0.6 < \Phi < 0.8$	Lamellar Axialitic growth
$0.6 < \Phi < 0.4$	2D planar structures
$\Phi < 0.4$	1D structures

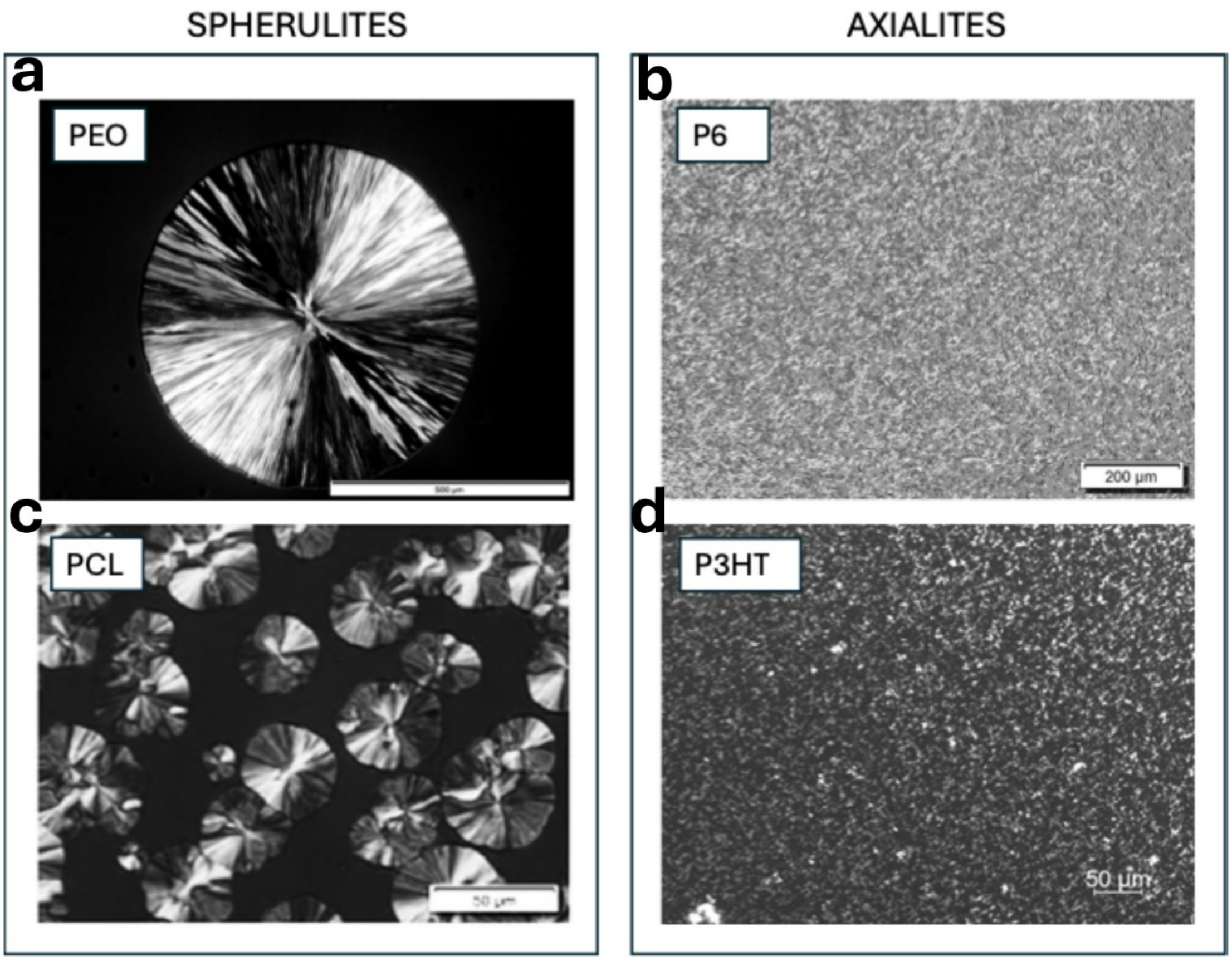

Figure S11 | Polarized optical microscopy (POM) images for different polymeric materials. Identification of the different birefringent features for PEO (**a**), P6 (**b**), PCL (**c**) and P3HT (**d**).

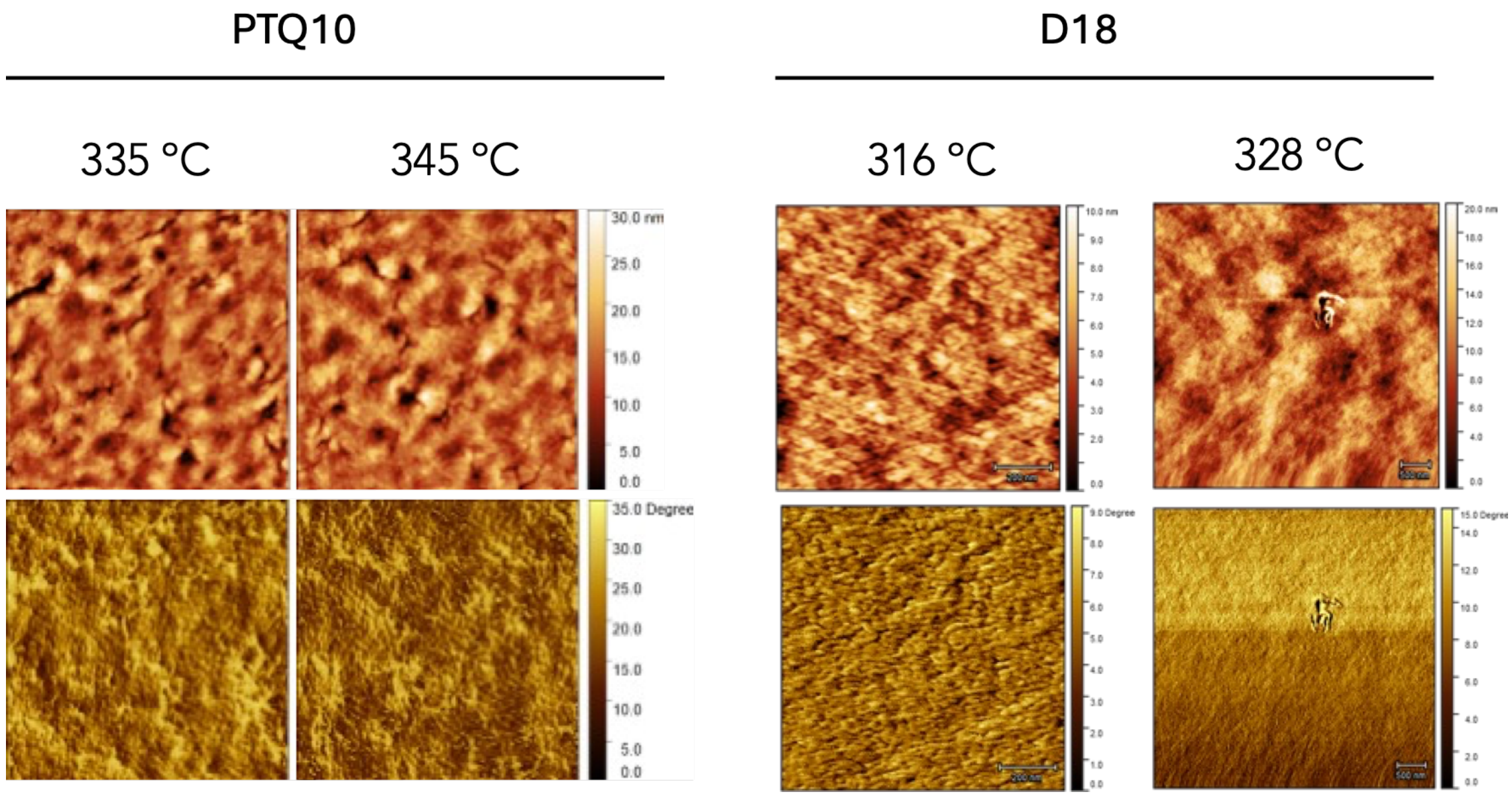

Figure S12. Atomic Force Microscopy topography (top) and phase-contrast (bottom) images for D18 and PTQ10 supported onto Flash DSC chips and isothermally crystallized at the indicated temperatures

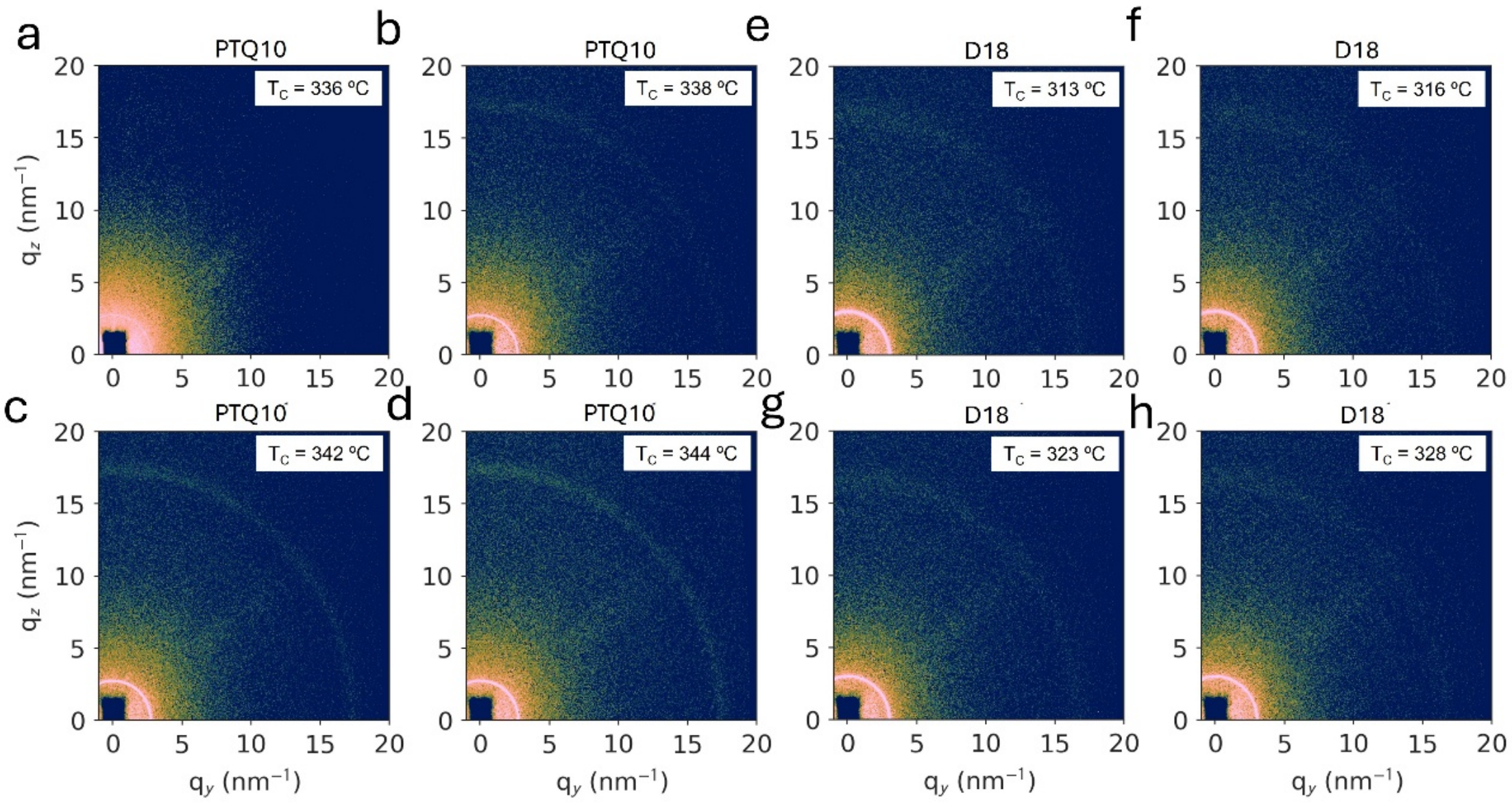

Figure S13. Room temperature 2D WAXS detector images (in reciprocal space units) of FSC isothermally crystallized

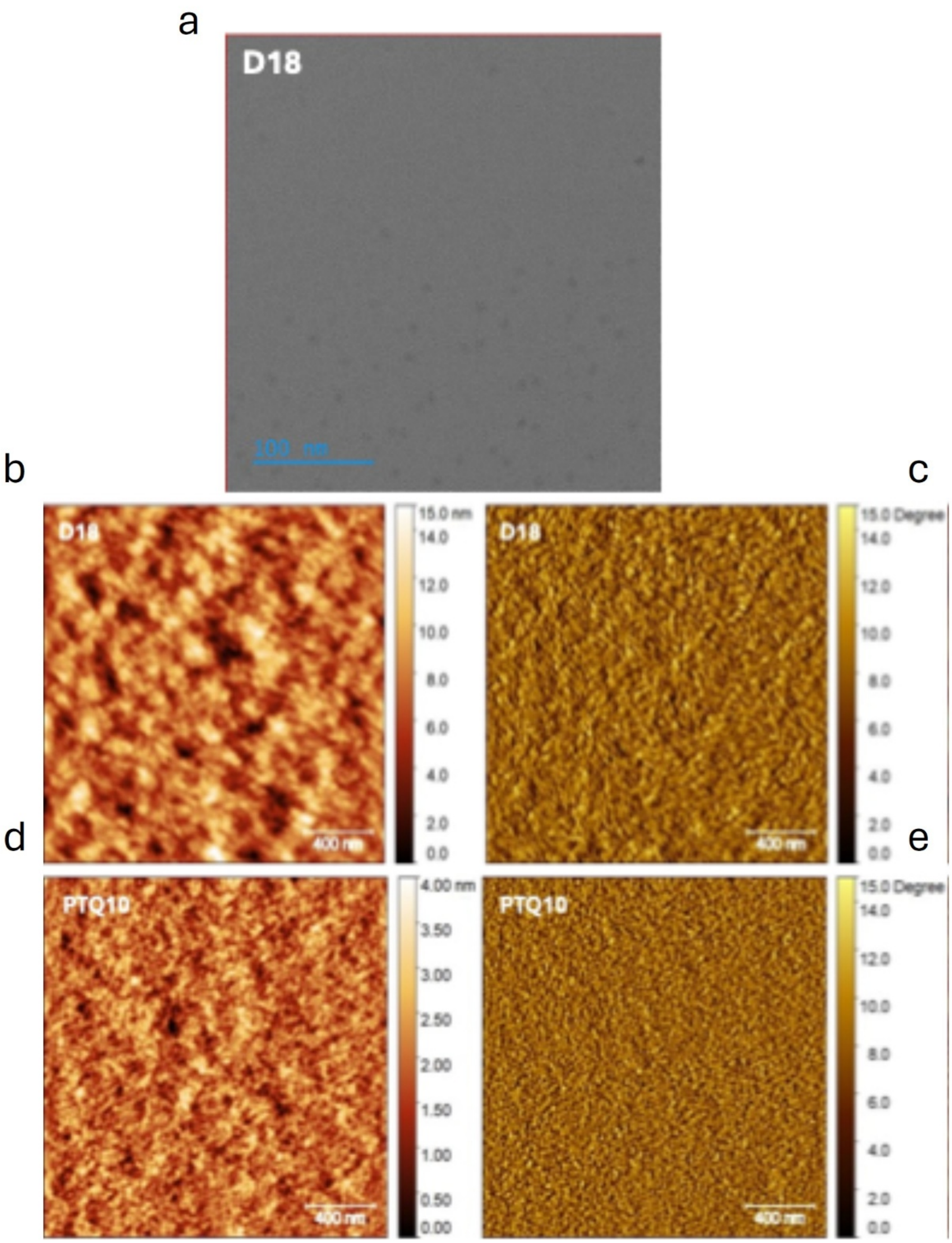

Figure S14. HR-TEM image for spun cast D18 films (a). Representative AFM topography (b) and phase contrast (c) images for spun cast D18 film. Representative AFM topography (d) and phase contrast (e) images for spun cast PTQ10 film.

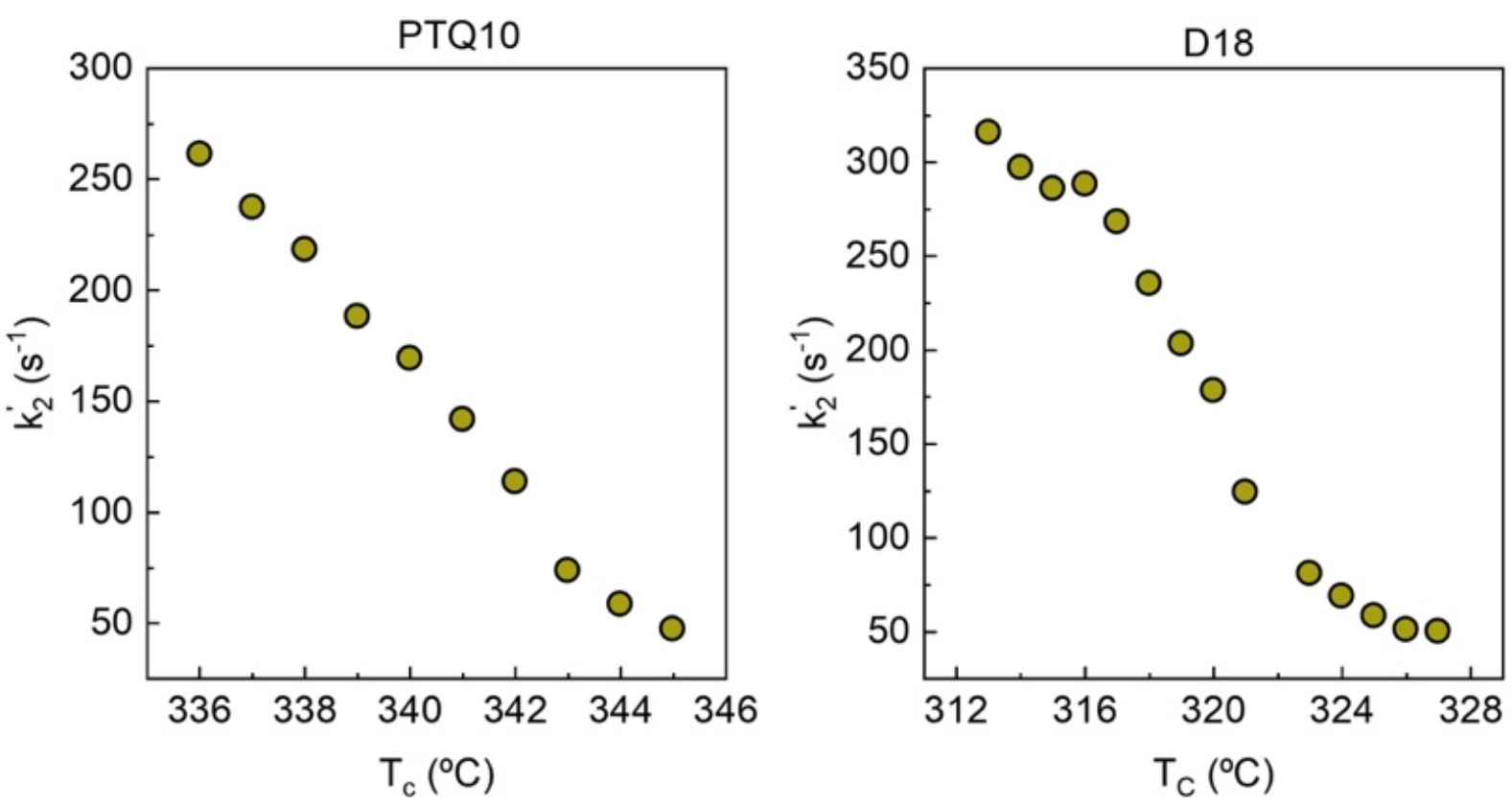

Figure S15. Dependence of the k_2' rate constant with the crystallization temperature, T_c.

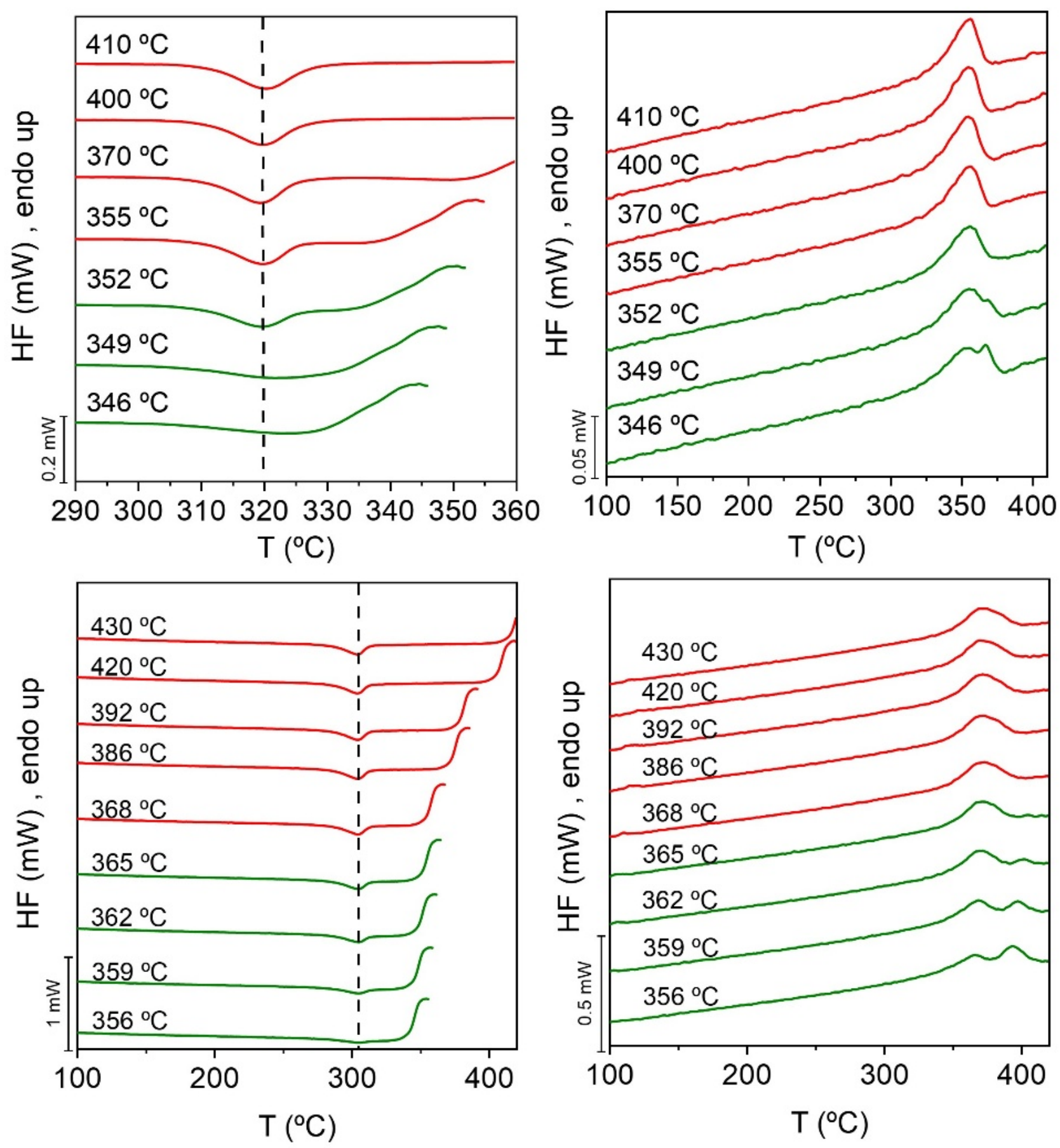

Figure S16. Flash DSC results of self-nucleation experiments conducted on PQT10 (top panes) and D18 (bottom panels) samples: (left panels) cooling traces after heating at different T_S; (right panels) reheating traces after cooling from different T_S;

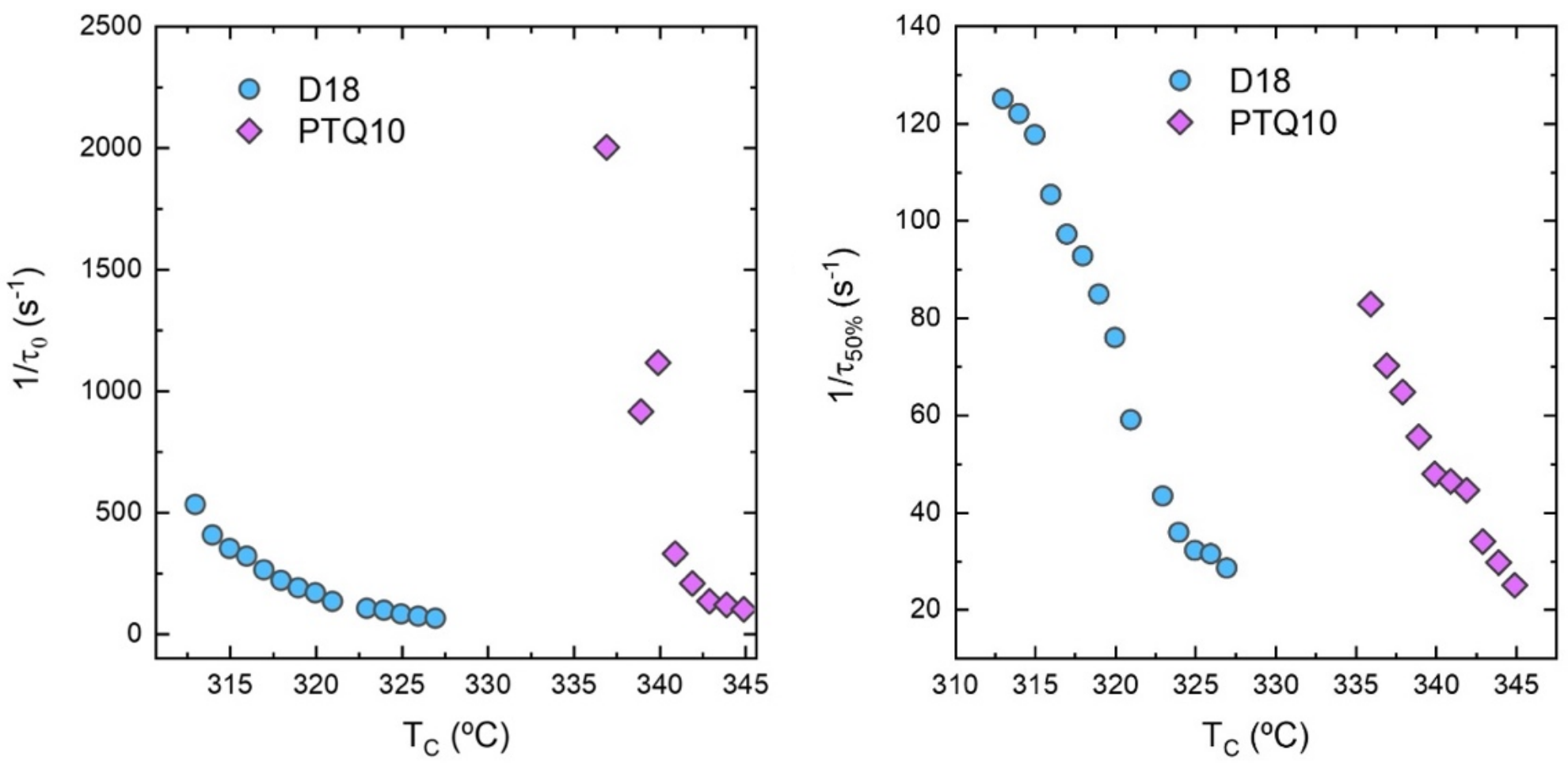

Figure S17. Dependence of the primary nucleation rate (as $1/t_0$) vs T_c and experimental values of the inverse of crystallization times ($1/\tau_{50\%}$)

References

(1) Rech, J. J.; Neu, J.; Qin, Y.; Samson, S.; Shanahan, J.; Josey, R. F.; Ade, H.; You, W. Designing Simple Conjugated Polymers for Scalable and Efficient Organic Solar Cells. *ChemSusChem* **2021**, *14* (17), 3561–3568. https://doi.org/10.1002/cssc.202100910.

(2) Olmedo-Martínez, J. L.; Pastorio, M.; Gabirondo, E.; Lorenzetti, A.; Sardon, H.; Mecerreyes, D.; Müller, A. J. Polyether Single and Double Crystalline Blends and the Effect of Lithium Salt on Their Crystallinity and Ionic Conductivity. *Polymers (Basel).* **2021**, *13* (13). https://doi.org/10.3390/polym13132097.

(3) Balsamo, V.; Urdaneta, N.; Pérez, L.; Carrizales, P.; Abetz, V.; Müller, A. J. Effect of the Polyethylene Confinement and Topology on Its Crystallisation within Semicrystalline ABC Triblock Copolymers. *Eur. Polym. J.* **2004**, *40* (6), 1033–1049. https://doi.org/10.1016/j.eurpolymj.2004.01.009.

(4) Berlic, C.; Barna, V. *SPORADIC POLYMER CRYSTALLIZATION IN THE N-DIMENSIONAL SPACE*; Vol. 11.

(5) Müller, A. J.; Balsamo, V.; Arnal, M. L. Nucleation and Crystallization in Diblock and Triblock Copolymers. *Advances in Polymer Science*. 2005, pp 1–63. https://doi.org/10.1007/12_001.

(6) Olmedo-Martínez, J. L.; Porcarelli, L.; Porcarelli, L.; Alegría, Á.; Alegría, Á.; Mecerreyes, D.; Müller, A. J. High Lithium Conductivity of Miscible Poly(Ethylene Oxide)/Methacrylic Sulfonamide Anionic Polyelectrolyte Polymer Blends. *Macromolecules* **2020**, *53* (11), 4442–4453. https://doi.org/10.1021/acs.macromol.0c00703.

(7) Fernández-Tena, A.; Pérez-Camargo, R. A.; Coulembier, O.; Sangroniz, L.; Aranburu, N.; Guerrica-Echevarria, G.; Liu, G.; Wang, D.; Cavallo, D.; Müller, A. J. Effect of Molecular Weight on the Crystallization and Melt Memory of Poly(ϵ-Caprolactone) (PCL). *Macromolecules* **2023**, *56* (12), 4602–4620. https://doi.org/10.1021/acs.macromol.3c00234.

(8) Olmedo-Martínez, J. L.; Pastorio, M.; Gabirondo, E.; Lorenzetti, A.; Sardon, H.; Mecerreyes, D.; Müller, A. J. Polyether Single and Double Crystalline Blends and the Effect of Lithium Salt on Their Crystallinity and Ionic Conductivity. *Polymers (Basel).* **2021**, *13* (13). https://doi.org/10.3390/polym13132097.

(9) Brinkmann, M. Structure and Morphology Control in Thin Films of Regioregular Poly(3-Hexylthiophene). *Journal of Polymer Science, Part B: Polymer Physics*. September 1, 2011, pp 1218–1233. https://doi.org/10.1002/polb.22310.

(10) Kuebler, J.; Loosbrock, T.; Strzalka, J.; Fernandez-Ballester, L. Direct Observation of Two-Step, Stratified Crystallization and Morphology in Conjugated Polymer Thin Films. *Macromolecules* **2023**, *56* (8), 3083–3094. https://doi.org/10.1021/acs.macromol.2c02439.
